# Deep sequencing-based characterization of transcriptome of trifoliate orange (*Poncirus trifoliata* (L.) Raf.) in response to cold stress

**DOI:** 10.1186/s12864-015-1629-7

**Published:** 2015-07-29

**Authors:** Min Wang, Xiaona Zhang, Ji-Hong Liu

**Affiliations:** Key Laboratory of Horticultural Plant Biology (MOE), College of Horticulture and Forestry Science, Huazhong Agricultural University, 430070 Wuhan, China

**Keywords:** *Poncirus trifoliata*, RNA-seq, Cold stress, Transcriptome profiling, Digital gene expression, Citrus

## Abstract

**Background:**

Trifoliate orange (*Poncirus trifoliata* (L.) Raf.) is extremely cold hardy after a full acclimation; however the underlying molecular mechanisms underlying this economically valuable trait remain poorly understood. In this study, global transcriptome profiles of trifoliate orange under cold conditions (4 °C) over a time course were generated by high-throughput sequencing.

**Results:**

More than 68 million high-quality reads were produced and assembled into a non-redundant data of 77,292 unigenes with an average length of 1112 bp (N50 = 1778 bp). Of these, 23,846 had significant sequence similarity to known genes and these were assigned to 61 gene ontology (GO) categories and 25 clusters of orthologous groups (COG) involved in 128 KEGG pathways. Sequences derived from cold-treated and control plants were mapped to the assembled transcriptome, resulting in the identification of 5549 differentially expressed genes (DEGs). These comprised 600 (462 up-regulated, 138 down-regulated), 2346 (1631 up-regulated, 715 down-regulated), and 5177 (2702 up-regulated, 2475 down-regulated) genes from the cold-treated samples at 6, 24 and 72 h, respectively. The accuracy of the RNA-seq derived transcript expression data was validated by analyzing the expression patterns of 17 DEGs by qPCR. Plant hormone signal transduction, plant-pathogen interaction, and secondary metabolism were the most significantly enriched GO categories amongst in the DEGs. A total of 60 transcription factors were shown to be cold responsive. In addition, a number of genes involved in the catabolism and signaling of hormones, such as abscisic acid, ethylene and gibberellin, were affected by the cold stress. Meanwhile, levels of putrescine progressively increased under cold, which was consistent with up-regulation of an arginine decarboxylase gene.

**Conclusions:**

This dataset provides valuable information regarding the trifoliate orange transcriptome changes in response to cold stress and may help guide future identification and functional analysis of genes that are importnatn for enhancing cold hardiness.

**Electronic supplementary material:**

The online version of this article (doi:10.1186/s12864-015-1629-7) contains supplementary material, which is available to authorized users.

## Background

Plants are frequently challenged by various environmental stresses, among which cold temperatures are one of the major factors that adversely influence plant growth and development, crop yield potential and geographic distribution [1]. Plants have evolved a diverse set of adaptive mechanisms in order to withstand cold stresses, one of these is acquired freezing tolerance following prior exposure to non-freezing low temperature, a phenomenon known as cold acclimation (CA) [2]. Studies of the mechanisms underlying CA-mediated improvement of freezing tolerance have suggested the importance of a wide range of physiological, biochemical, cellular and molecular processes, and these have been associated with modulation of gene transcription [3].

Significant progress has been made in identifying key components of the cold stress signaling network. Of particular note was the identification and characterization of C-repeat (CRT)-binding factors (CBFs), also known as the dehydration-responsive element-binding factors 1 (DREB1s) [4], which have been reported to be conserved among dicots and monocots. CBF genes play critical roles in mediating cold stress response by regulating a spectrum of cold-regulated (COR) genes, collectively called the CBF regulon, through binding to the *cis*-acting element (CRT/DRE) within their promoters [4, 5]. Regulation of COR genes by CBFs constitutes the predominant cold signaling pathway in plants. In addition, characterization of either positive or negative regulators of CBF genes, including *ICE1* (Inducer of CBF Expression 1), *HOS1* (High Expression of Osmotically Responsive Gene 1), and *MYB15* has provided a more complete understanding of the complexity of CBF-mediated cold signaling [6–8].

The expression patterns of large numbers of cold-responsive genes, including both regulatory genes and structural genes, are altered during exposure to cold [5]. For example, in *Arabidopsis thaliana* approximately 1000 genes were shown to exhibit different expression under cold stress, while more than 2 % of wheat genome were reported to show altered expression in response to cold [9, 10]. Several other studies have reported similar changes in gene expression patterns in various plant species [11–13]. Although increasing numbers of genes involved in cold responses are being identified and characterized in an array of plant species, the proportions are still small relative to observed genome wide changes, and most genes involved in this process have yet to be functionally characterized. On the other hand, plants have also developed a CBF-independent pathway for adapting to cold stress, which is corroborated by the finding that only 12 % of cold-responsive genes are likely under the control of CBFs [14]. However, this CBF-independent pathway has not been well characterized.

Genome-wide transcriptome analysis represents a potentially valuable strategy for elucidating the breadth of molecular mechanisms underlying physiological processes and it can substantially increase the efficiency of identifying genes of interest. Recent developments in high-throughput DNA sequencing technologies and associated analytical approaches, such as RNA-Seq, allow the generation of large-scale transcriptome data for both model and non-model species [15]. Over the last few years, deep sequencing using Illumina-based RNA-Seq has been increasingly applied to capture an overview of RNA transcript profiles in a range of plant species following exposure to diverse and adverse environmental conditions, including high salinity, drought, cold temperatures, and pathogen attack [11–13, 16–18]. For example, Illumina sequencing-based snapshots of the transcriptomes of non-model species, such as *Anthrurim andraeanum*, *Camellia sinensis* and *Lilium lancifolium* under cold treatment have been reported, together with identification of many differentially expressed genes (DEGs) [11–13]. RNA-Seq has thus been proven to act as a powerful approach for creating transcriptomic data for non-model plants, particularly those without substantial existing sequence data [19].

Trifoliate orange (*Poncirus trifoliata* (L.) Raf.), a diploid (2n = 2× = 18) with high degree of heterozygosity, is most closely related to *Citrus* spp., the genome of which has been sequenced [20]. One of its notable attributes is the striking cold hardiness after a period of CA. To date, several cold-responsive *P. trifoliata* genes have been isolated by homology-based cloning and low-throughput cDNA library screening. One such study described the characterization of cDNAs corresponding to eight up-regulated and six down-regulated *P. trifoliata* genes following a gradual CA temperature regime, using mRNA differential display-reverse transcription (DDRT)-PCR [21]. Additionally, suppression subtractive hybridization (SSH) has been used to screen cDNA libraries, resulting in the identification of non-redundant differentially expressed sequence tags (ESTs) [22, 23]. Based on these studies, several *P. trifoliata* stress-responsive regulatory or structural genes, such as *PtADC*, and *PtrbHLH*, have been functionally characterized and shown to play key roles in cold tolerance [24, 25]. However, such analytical approaches have not provided a global overview of the molecular mechanisms underlying the CA-mediated cold tolerance in *P. trifoliata*. In this current study, a comprehensive transcriptome analysis of trifoliate orange under cold stress was performed, using the Illumina-based paired-end deep sequencing platform. More than 6.1 million pairs of high-quality sequence were assembled and annotated, and large sets associated with transcripts involved in diverse metabolic processes and signaling pathways were identified. In addition, a total of 5549 predicted genes with regulatory or protective roles were revealed to be either up- or down-regulated by cold at different time points. Exposure to cold temperatures resulted in an increase in the abundance of transcripts associated with several metabolic pathways and the expression data further suggested the involvement of both the CBF-dependent and independent pathways in the cold responses. The RNA-seq and digital expression profiling described here have yielded valuable insights into the understanding the molecular events related to cold responses in trifoliate orange and the dataset represents a significant resources for targeting genes in future breeding efforts.

## Results

### Transcriptome sequencing and *de novo* assembly

**Fig. 1:**
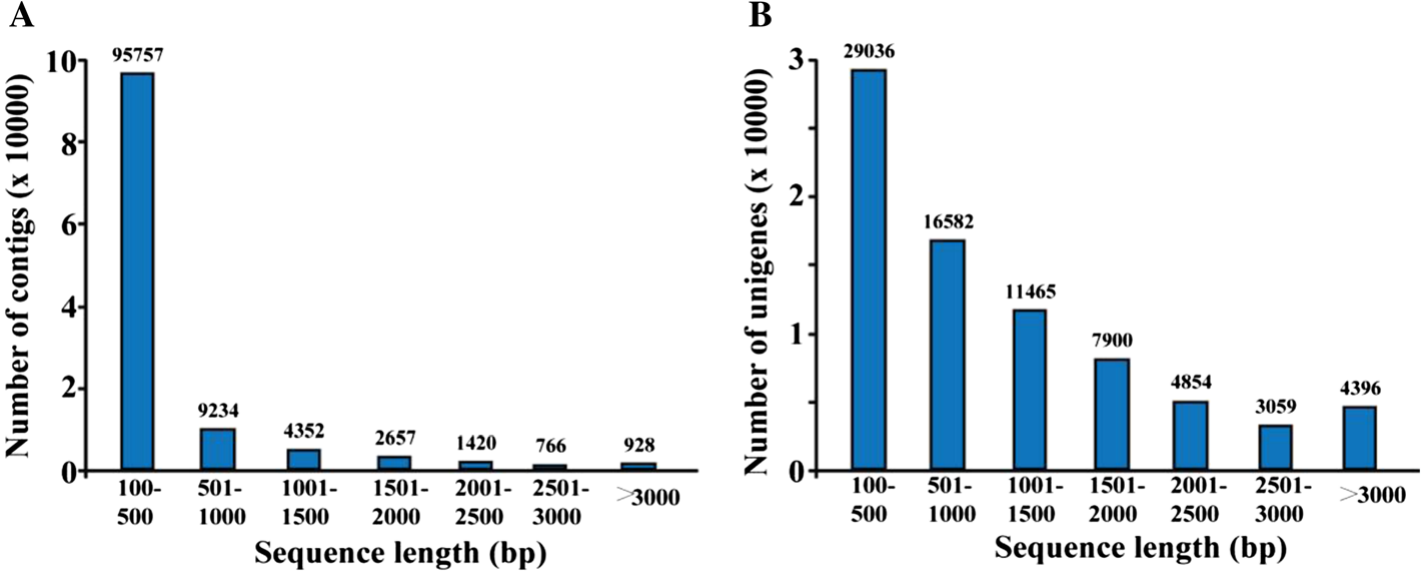
Length distributions of contigs (**a**) and unigenes (**b**) in trifoliate orange transcriptome

A cDNA library was constructed using equal amounts of RNA extracted from *P. trifoliata* seedlings that had been exposed to various stresses, including cold temperatures (4 °C), high salinity and drought. To characterize the trifoliate orange transcriptome, the cDNA library was subjected to paired-end (PE) read sequencing using the Illumina HiSeq2000 platform. After removing reads of low quality, adaptor sequences or reads with >5 % ambiguous nucleotides, we got a total of 68,041,582 clean PE reads consisting of 6,123,742,380 nucleotides (5.84 Gb), with an average GC content of 45.2 %. These high-quality reads were then *de novo* assembled using the Trinity program [26], resulting in 115,114 contigs with an average length of 372 bp and an N50 length of 776 bp (Table 1). Based on the PE sequence information, the contigs were further assembled to give 77,292 unigenes, which accounted for 85,969,539 nucleotides (82.0 Mb), with an average length of 1112 bp. Of these unigenes, 48,256 (62.5 %) were longer than 500 bp, 31,674 (41.0 %) were longer than 1000 bp, 20,209 (26.2 %) were longer than 1500 bp and 12,309 (15.9 %) were longer than 2000 bp (Fig. 1). Further analysis showed that sequencing depth of the unigenes ranged from 0.0175 to 93,708, with an average of 49.57, while the number of reads uniquely mapped to a unigene ranged from 1 to 1,675,941. The transcriptome data have been deposited at DDBJ/EMBL/GenBank under the accession number of GCVN00000000.

**Table 1:** Summary of *P. trifoliata* RNA-Seq data

	High-quality sequences (n)	Total bases (base pairs, bp)	Average length (bp)	N50 (bp)
Reads	68,041,582	6,123,742,380	2*90	-
Contigs	115,114	42,846,307	372	776
Unigenes	77,292	85,969,539	1112	1778

Gene expression levels were calculated based on FPKM (fragments per kb per million fragments) method [26], and among the unigenes, 28,393 (36.7 %) and 65,372 (84.5 %) had FPKM values smaller than 1.0 and 10.0, respectively, whereas only 1241 (1.6 %) had FPKM values > 100. This suggests that most of the unigenes were expressed at low levels.

### Functional annotation of unigenes

**Fig. 2:**
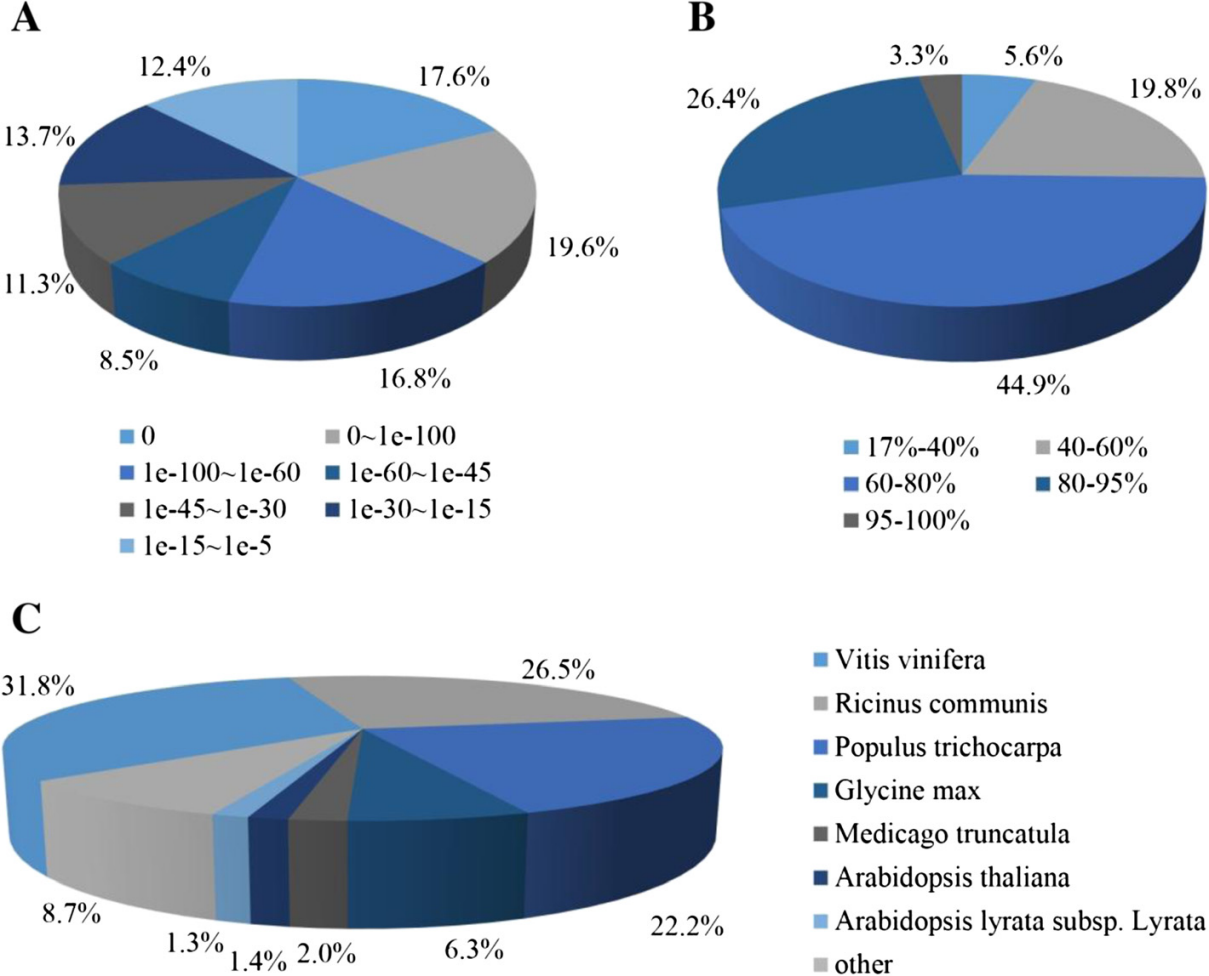
Characteristics of homology search of unigenes against NCBI non-redundant (Nr) database. **a**. *E*-value distribution of BLAST hits for each unique sequence with a cut-off *E*-value of 1e^−5^. **b**. Similarity distribution of top BLAST hits for each unigene. **c**. Species distribution of the top BLAST hits for each unigene with a cut-off *E*-value of 1e^−5^

The assembled unigene sequences were used to search public databases, including the NCBI non-redundant protein database (nr), the Swissprot protein database, the Gene Ontology (GO) database, the Clusters of Orthologous Groups (COGs) database, and Kyoto Encyclopedia of Genes and Genomes (KEGG) database, using the BLASTX algorithm with an *E*-value threshold of 1e^−5^. In total, 59,777 (77.3 %) unigenes were matched to a sequence in at least one of the above-mentioned databases (Table 2) and 58,001 and 36,445 unigenes had significant hits (*E*-value < 1e^−5^) in the Nr and Swissprot non-redundant protein databases, respectively. In terms of *E*-value distribution, 37.4 % of the homologs ranged between 1e^−5^ and 1e^−45^, while a majority of the sequences (62.5 %) showed a threshold e-value < 1e^−45^ indicating strong homology (Fig. 2a). Among the unigenes annotated using the Nr database, 74.6 % had more than 60 % similarity with the corresponding gene sequence (Fig. 2b). Overall, the unigene sequences exhibited most similar BLASTx matches to gene sequences from *Vitis vinifera* (31.8 %), followed by those from *Ricinus communis* (26.5 %), *Populus trichocarpa* (22.2 %), *Glycine max* (6.3 %), *Medicago truncatula* (2.0 %) and *A. thaliana* (1.4 %) genes (Fig. 2c).

**Table 2:** List of *Poncirus trifoliata* transcriptome annotations

Public database	No. of unigene hits	Percentage (%)
Nt	53,702	69.5
Nr	58,001	75.0
SwissProt	36,445	47.2
GO	34,747	45.0
COG	23,846	30.9
KEGG	45,819	59.3
ALL	59,777	77.3

Nt: Nucleotide database; Nr: Non-redundant protein sequence database

SwissProt: Swiss-Prot protein sequence database; GO: Gene Onotology database

COG; Cluster of Orthologous Groups of proteins

KEGG: Kyoto Encyclopedia of Genes and Genomes

**Fig. 3:**
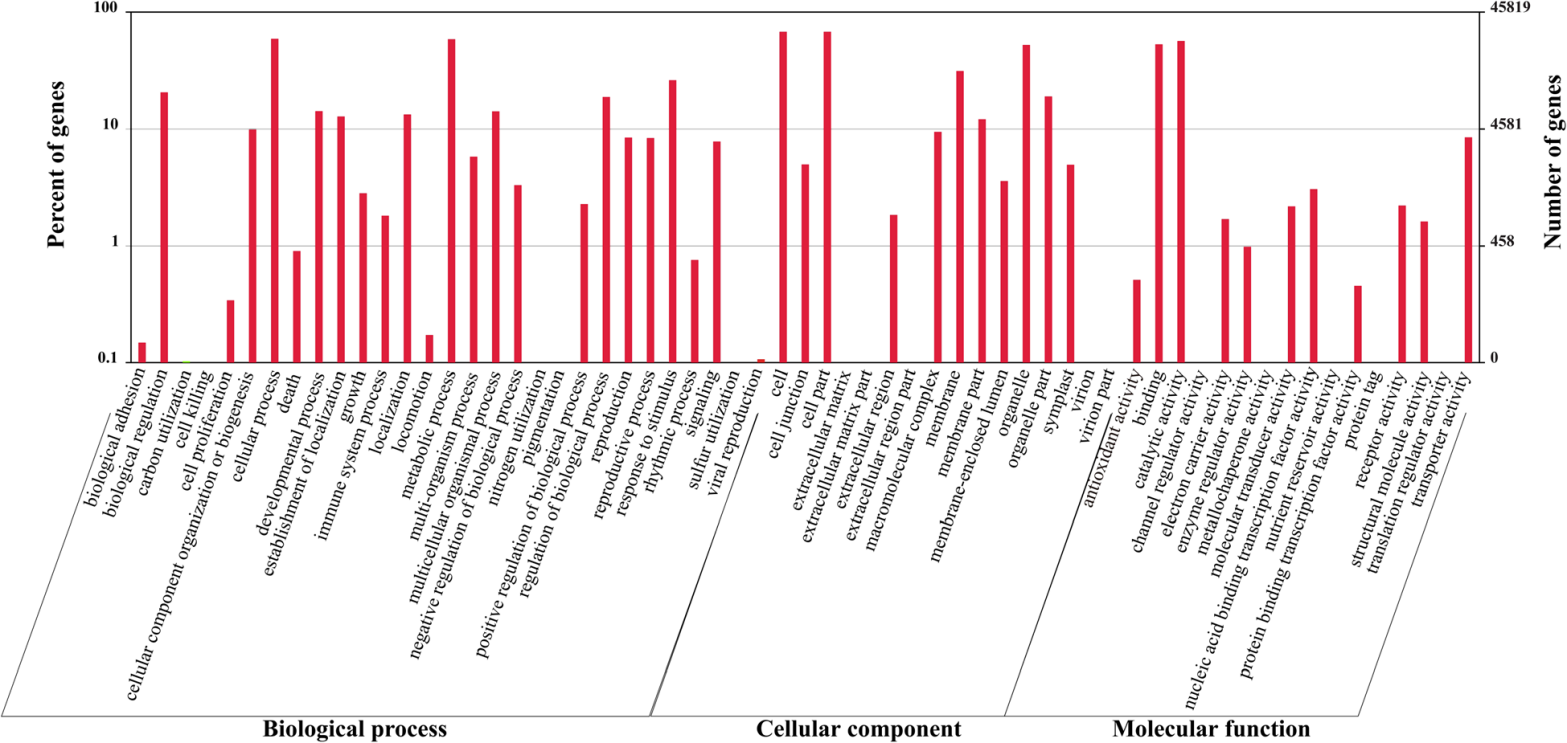
Histogram of Gene Ontology (GO) classifications. Percentages indicate the proportion of unigenes that have the relevant GO annotations, including biological process, cellular components and molecular function

GO analysis was widely used to assign putative gene function to uncharacterized sequences. We used the information from the Nr annotation together with the WEGO program [27] to categorize 34,747 unigenes into 61 functional groups (Fig. 3), and these were further divided among three GO terms: ‘including molecular function’, ‘cellular component’, and ‘biological process’ using BLAST2GO [28]. Most of the unigenes in the ‘molecular function’ category were sub-categorized into ‘catalytic activity’ (25,902) and ‘binding’ (24,250), followed by ‘transporter activity’ (3890) and ‘nucleic acid binding transcription factor activity’ (1400). In the ‘cellular component’ category, the GO terms ‘cell’ (31,049), ‘cell part’ (31,049), ‘organelle part’ (23,958), and ‘membrane’ (14,312) predominated, while ‘cellular process’ (27,062), ‘metabolic process’ (26,810), and ‘response to stimulus’ (12,031) were the three most represented GO terms in the ‘biological process’ category.

**Fig. 4:**
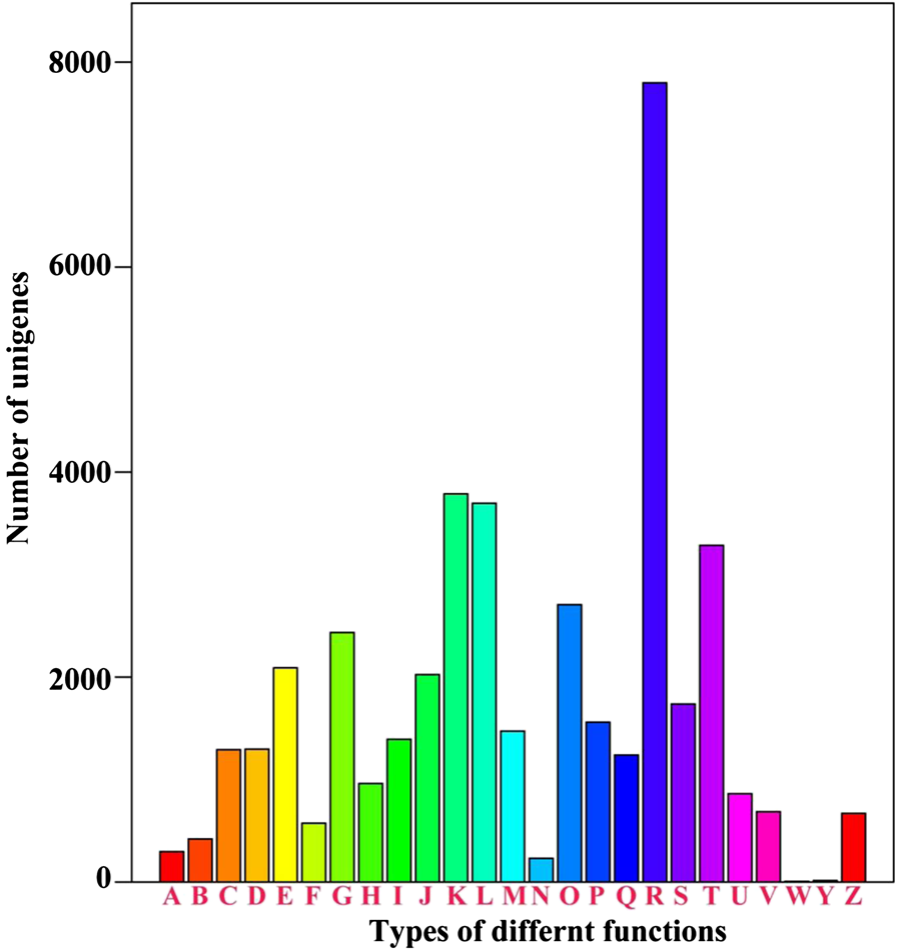
Distribution of genes with COG functional classification in the transcriptome. A total of 23,846 sequences have a COG classification among 25 categories. A: RNA processing and modification; B: Chromatin structure and dynamics; C: Energy production and conversion; D: Cell cycle control, cell division, chromoso; E: Amino acid transport and metabolism; F: Nucleotide transport and metabolism; G: Carbohydrate transport and metabolism; H: Coenzyme transport and metabolism; I: Lipid transport and metabolism; J: Translation, ribosomal structure and biogenesis; K: Transcription; L: Replication, recombination and repair; M: Cell wall/membrane/envelope biogenesis; N: Cell motility; O: Posttranslational modification, protein turnover, chaperones; P: Inorganic ion transport and metabolism; Q: Secondary metabolites biosynthesis, transport and catabolism; R: General function prediction only; S: Function unknown; T: Signal transduction mechanisms; U: Intracellular trafficking, secretion, and vesicular transport; V: Defense mechanisms; W: Extracellular structures; Y: Nuclear structure; Z: Cytoskeleton

We then performed a phylogenetic classification of the trifoliate orange sequences using the Cluster of Orthologous Groups (COG) database, which is composed of protein sequences derived from the genomes of bacteria, plants, and animals. Each protein in the COG database is assumed to have evolved from an ancestral protein. On the basis of sequence similarities, of the 36,445 SwissProt sequence matches to the trifoliate orange sequences, 23,846 unigenes could be grouped into 25 COG categories (Fig. 4), Of these ‘general function prediction only’ (7797; 32.7 %) represented the largest class, followed by ‘transcription’ (3786; 15.9 %), ‘replication, recombination and repair’ (3695; 15.5 %), and ‘signal transduction mechanisms’ (3283; 13.8 %), whereas 1736 (5.8 %) were assigned to the category of ‘function unknown’ category. In addition, only a few unigenes grouped in the ‘extracellular structures’ (7, 0.03 %) and ‘nuclear structure’ (14, 0.06 %) categories.

When the assembled unigenes were analyzed using the BLASTX function of the KEGG database using BLASTX. As a result, 45,819 (59.3 %) mapped to 128 KEGG pathways (Additional file 1), of which ‘metabolic pathway’ (7987; 23.0 %), ‘biosynthesis of secondary metabolites’ (3923; 11.3 %), ‘plant-pathogen interaction’ (2111; 6.1 %), ‘plant hormone signal transduction’ (1693; 4.9 %) and ‘RNA transport’ (1067; 3.1 %) were the most abundant groups. Interestingly, we noted that a large number of unigenes involved in the biosynthesis of primary metabolites, such as starch, sucrose, arginine, and proline, all of which have been reported to be associated with stress tolerance, were also enriched in the dataset (Additional file 2).

**Fig. 5:**
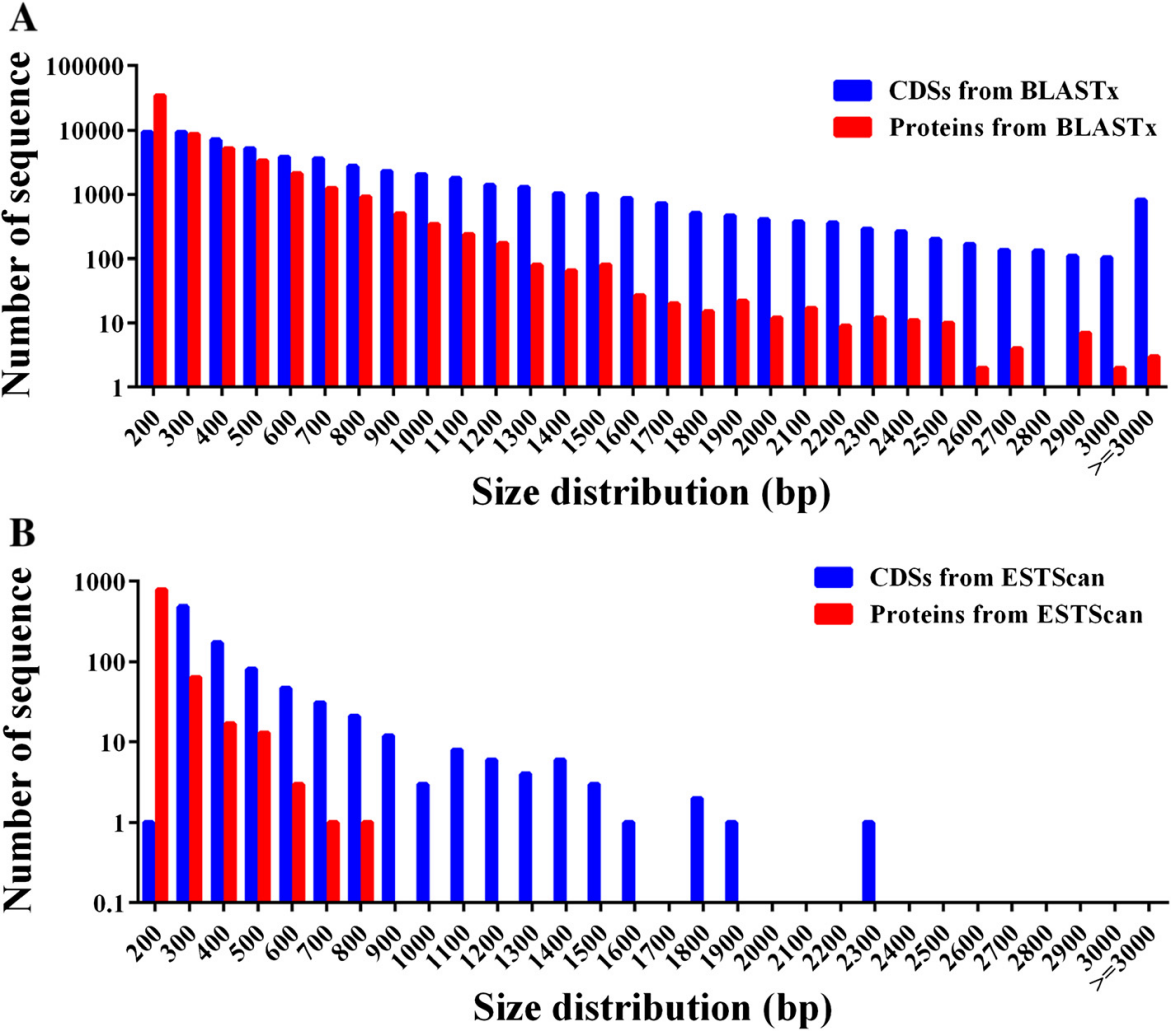
Coding sequence (CDS) of unigenes predicted by BLASTx and ESTScan. The size distribution of the CDSs and proteins based on BLASTx (**a**) and ESTScan (**b**)

*P. trifoliata* protein-coding sequences were deduced using BLASTx and the unigene sequences to interrogate the Nr, SwissProt, KEGG, and COG, using BLASTx protein databases, resulting in a total of 57,934 predicted coding sequences (CDSs). Among the unigenes with CDSs, 49.6 % were 500–2000 bp, while 44.6 % were < 500 bp, and 811 were > 3000 bp (Fig. 5). A total of 889 CDSs did not have a match in any of the protein databases using ESTScan program [29].

### Identification of differentially expressed genes (DEGs)

**Fig. 6:**
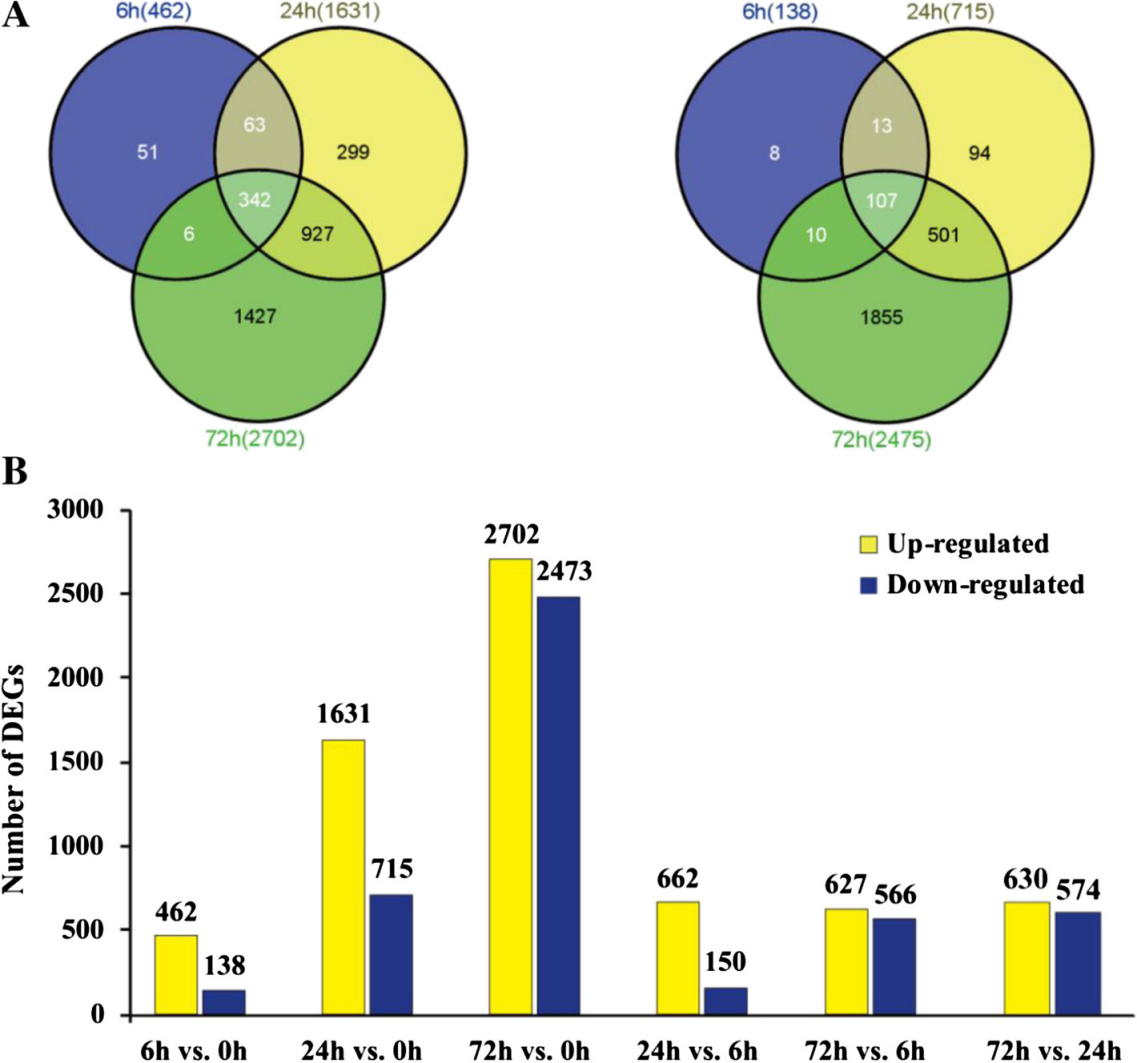
Differentially expressed genes (DEGs) of *Poncirus trifoliata* under cold stress. A-B. Venn maps of up-regulated (**a**) and down-regulated genes (**b**). C. The number of up-regulated and down-regulated DEGs compared between different time points under cold treatment at 0, 6, 24 and 72 h

In order to gain a global view of transcript expression in trifoliate orange in response to cold stress and to identify cold-responsive genes, we constructed four cDNA libraries from plants either grown under normal conditions or exposed to a cold treatment (4 °C for 6, 24, or 72 h). After data filtering, at least 7.1 million 49 nt single-end clean reads were obtained for each library, which represented ~99.3 % of the raw data (Table 3). An analysis of sequencing saturation indicated that the output of cleaned reads was sufficient to identify DEGs (Additional file 3). The filtered cleaned reads were then mapped onto the assembled *P. trifoliata* transcriptome using SOAPaligner/soap2 software [30]. The uniquely matched reads, which ranged from 45.5 % to 49.3 % of the total cleaned reads, were subsequently used to assess relative expression levels (Table 3). Each transcript had a range of read coverage, but more than 40 % of the detected unigenes had > 50 % coverage (data not shown). According to the criteria used for identifying DEGs, including low false discovery rate (FDR, less than 10^−3^) and |log_2_FPKM|> 1.0, a total of 5549 unigenes were considered to be differentially expressed between control and cold treated plants, with 600 at in the 6 h sample (462 up-regulated, 138 down-regulated), 2346 in the 24 h sample (1631 up-regulated, 715 down-regulated), and 5475 in the 72 h sample (2702 up-regulated, 2473 down-regulated) (Fig. 6a, b, Additional file 4 and Additional file 2). When the DEGs at different time points were evaluated (Fig. 6c), we observed that their numbers increased with longer cold treatments, and that the number of DEGs between two different cold treatment samples (24 vs 6, 72 vs. 6 and 72 vs. 24) was relatively small.

**Table 3:** Sequencing and read mapping of the differentially expressed genes (DEGs)

Summary	0 h	6 h	24 h	72 h	Average
Raw reads	7,164,446	7,325,695	7,563,619	7,212,252	7,316,503
Clean reads	7,113,843	7,271,861	7,515,414	7,163,347	7,266,116
Total base pairs	348,578,307	356,321,189	368,255,286	351,004,003	356,039,696
Clean Reads (%)	99.29	99.27	99.36	99.32	99.31
Mapped reads	6,621,141	6,727,919	6,879,216	6,467,083	6,467,087
Mapped reads* (%)	93.07	92.52	91.53	90.28	91.85
Perfect match	5,583,419	5,707,629	5,813,295	5,505,742	5,652,521
Perfect match* (%)	78.49	78.49	77.35	76.83	77.79
<=2 Mismatch	1,037,722	1,020,290	1,065,921	961,341	1,021,319
<=2 Mismatch* (%)	14.59	14.03	14.18	13.42	14.06
Unique match	3,117,820	3,309,503	3,456,940	3,290,477	3,293,685
Unique match* (%)	43.83	45.51	46.00	45.93	45.33
Multi-Position match	3,503,321	3,418,416	3,422,276	3,176,606	3,380,155
Multi-Position match	49.25	47.01	45.54	44.35	46.54
Unmapped reads	492,702	543,942	636,198	696,264	592,277
Unmapped reads* (%)	6.93	7.48	8.47	9.72	8.15

### Gene Ontology clustering of DEGs

The DEGs were first mapped to the three main categories of the GO database: ‘molecular function’, ‘cellular component’ and ‘biological process’. Significantly enriched GO terms then determined using a hypergeometric test to identify functional clusters and biochemical pathways (corrected *P*-value). In the ‘biological process’ category, 18 GO terms were significantly enriched in the 6 h cold treatment sample (Additional file 5), of which the GO terms ‘response to stimulus’, ‘response to organic substance’, ‘response to stress’, ‘response to carbohydrate stimulus’, ‘response to chemical stimulus’ and ‘response to hormone stimulus’ were most highly enriched. We interpreted this to indicate an involvement of the genes mapping to these pathways in perception and transduction of the cold signal. The substantial up-regulation of certain kinases (CL6797.Contig3_All, Unigene16428_All, CL8004.Contig1_All) and TFs (Unigene20109_All, Unigene11424_All, Unigene14645_All, Unigene19023_All, Unigene21445_All) at 6 h was particularly notable. Also of interest was the fact that the GO terms ‘response to chitin’, ‘response to fungus’, ‘response to other organism’, ‘response to biotic stimulus’, ‘response to wounding’, ‘defense response’, ‘response to salicylic acid stimulus’ and ‘defense response to fungus’ were enriched in the 6 h sample, which suggests pathway crosstalk between biotic stress responses and the perception and transduction of cold induced signaling.

In the 24 h cold treatment sample the enriched GO terms in the ‘biological process’ category were similar to those at 6 h; however, more DEGs were identified in the various categories. Lastly, in the 72 h cold treatment sample, we observed that in addition to the stress-responsive categories, the two photosynthesis-related GO terms, ‘photosynthesis, light harvesting’ and ‘photosynthesis’ were over-represented. Interestingly, all of the DEGs related to photosynthesis were down-regulated upon exposure to the cold treatment, suggesting that the photosynthetic capacity was suppressed. In addition, the categories ‘RNA secondary structure unwinding’ and ‘translation’ were enriched in the 72 h sample, from which we infer that regulation of gene expression in response to cold temperatures may occur at the post-transcriptional and translational levels. Four unigenes enriched in ‘RNA secondary structure unwinding’ showed an increase in abundance, and three of these were annotated as glycine-rich RNA-binding protein s, which have been shown in *A. thaliana* to be involved in biotic or abiotic stress responses [31, 32]. The fourth gene was annotated as cellular nucleic acid binding protein.

### Pathways enrichment analysis of DEGs

Using the KEGG database, pathways displaying significant changes (Q value ≤ 0.05) in response to the cold treatment were identified (Table 4). In the 6 h cold treatment sample, 218 of the 600 DEGs were associated with KEGG pathways. This analysis revealed five significantly enriched pathways: ‘plant-pathogen interaction’, ‘plant hormone signal transduction’, ‘riboflavin metabolism’, ‘flavone and flavonol biosynthesis’ and ‘glycosyl phosphatidyl inositol-anchor biosynthesis’. In the 24 h cold treatment sample, enriched pathways included ‘plant-pathogen interaction’, ‘plant hormone signal transduction’, ‘ether lipid metabolism’, ‘GPI-anchor biosynthesis’, and ‘zeatin biosynthesis’ were significantly changed and more than 12 pathways were significantly changed after 72 h of cold treatment, among which ‘photosynthesis-antenna proteins’, ‘plant-pathogen interaction’, ‘photosynthesis’, ‘flavone and flavonol biosynthesis’ and ‘stilbenoid, diarylheptanoid and gingerol biosynthesis’ were the most significantly enriched. Interestingly, genes involved in the ‘plant-pathogen interaction’ pathway, such as *CDPK* (CL4696.Contig2_All), calcium-binding protein (Unigene18341_All, Unigene4416_All), and *WRKY* (CL3620.Contig1_All, Unigene11423_All) showed large changes in expression levels at all three points of the cold treatment (Table 4). Finally, the ‘plant hormone signal transduction’ pathway showed significant changes in all three samples, suggesting that hormone signaling is involved in the cold stress response of *P. trifoliata*.

**Table 4:** Significantly enriched gene pathways involving differentially expressed genes (DEGs) following the cold stress treatment

	Pathway	DEGs with pathway annotation	All genes with pathway annotation (34747)	Q value	Pathway ID
6 h vs 0 h	Plant-pathogen interaction	37	2111 (6.1 %)	8.66e-07	ko04626
	Plant hormone signal transduction	30	1693 (4.9 %)	9.60e-06	ko04075
	Riboflavin metabolism	5	101 (0.3 %)	9.47e-03	ko00740
	Flavone and flavonol biosynthesis	6	164 (0.5 %)	9.70e-03	ko00944
	Glycosylphosphatidylinositol(GPI)-anchor biosynthesis	6	229 (0.7 %)	4.23e-02	ko00563
24 h vs 0 h	Plant-pathogen interaction	125	2111 (6.1 %)	1.35e-16	ko04626
	Plant hormone signal transduction	86	1693 (4.9 %)	6.46e-08	ko04075
	Ether lipid metabolism	21	372 (1.1 %)	2.27e-02	ko00565
	Zeatin biosynthesis	15	245 (0.7 %)	3.55e-02	ko00908
	Glycosylphosphatidylinositol(GPI)-anchor biosynthesis	15	229 (0.7 %)	2.27e-02	ko00563
72 h vs 0 h	Photosynthesis - antenna proteins	13	26 (0.1 %)	1.12e-07	ko00196
	Plant-pathogen interaction	190	2111 (6.1 %)	6.43e-06	ko04626
	Photosynthesis	22	106 (0.3 %)	1.89e-05	ko00195
	Flavone and flavonol biosynthesis	25	164 (0.5 %)	8.39e-04	ko00944
	Diterpenoid biosynthesis	17	100 (0.3 %)	2.11e-03	ko00904
	Ether lipid metabolism	42	372 (1.1 %)	2.11e-03	ko00565
	Plant hormone signal transduction	142	1693 (4.9 %)	2.11e-03	ko04075
	Ribosome	46	431 (1.2 %)	3.27e-03	ko03010
	Cutin, suberine and wax biosynthesis	19	129 (0.4 %)	4.61e-03	ko00073
	Limonene and pinene degradation	33	328 (0.9 %)	4.37e-02	ko00903
	Endocytosis	61	664 (1.9 %)	1.58e-02	ko04144
	Stilbenoid, diarylheptanoid and gingerol biosynthesis	39	330 (1.0 %)	2.10e-03	ko00945

### Validation of RNA-seq based DEG results by quantitative real-time RT-PCR (qPCR)

**Fig. 7:**
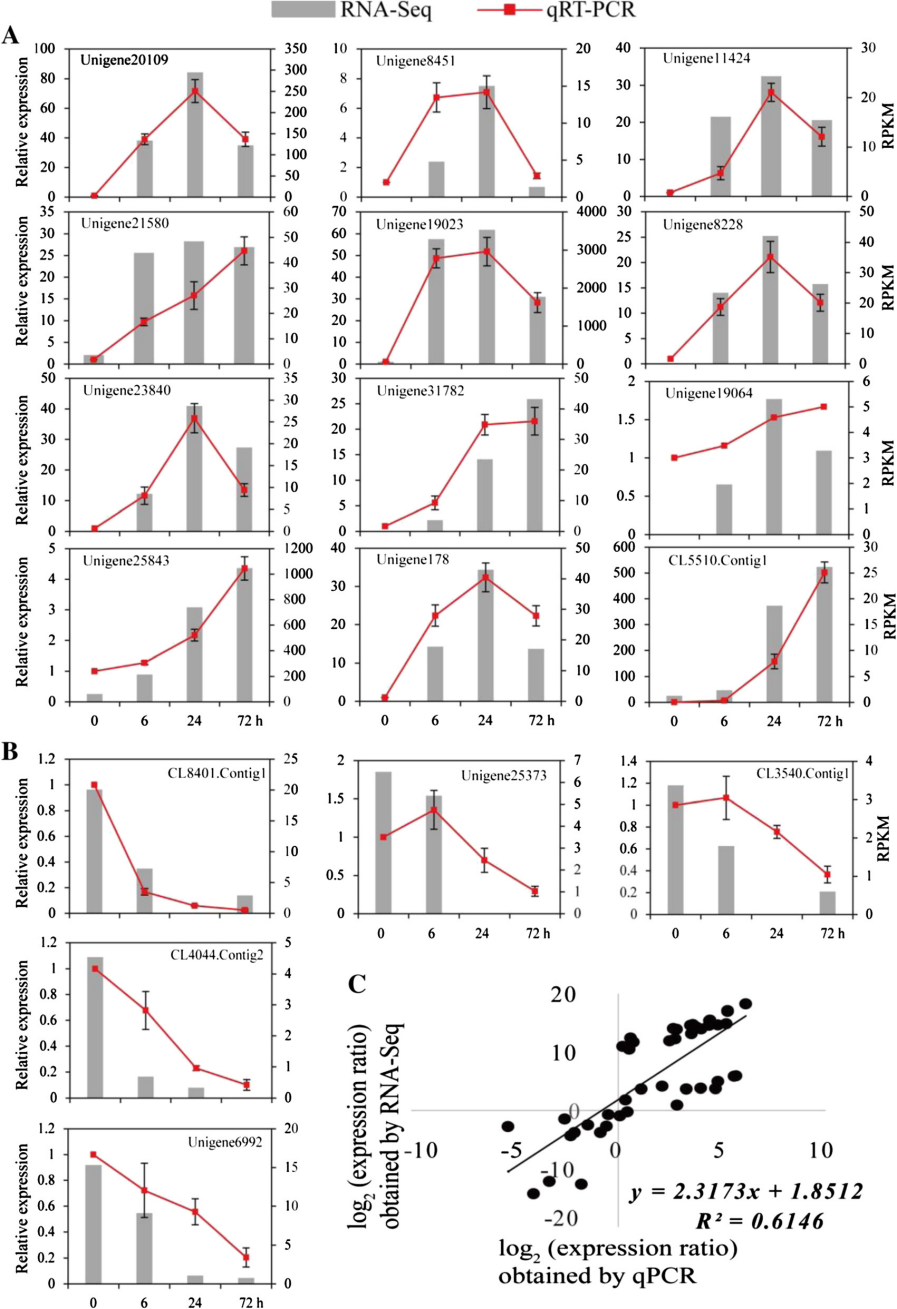
qPCR analysis of differentially expressed genes in *Poncirus trifoliata* under low temperature. (**a**, **b**) Transcript levels of 17 randomly selected DEGs, including 12 up-regulated (**a**) and 5 down-regulated (**b**). The Y-axis on the left shows the relative gene expression levels analyzed by qPCR (red lines), while Y-axis on the right shows corresponding expression data of RNA-seq (gray histogram). The X-axis represents the time (hours) of 4 °C treatment. The bars represent SE (n = 3). (**c**) Comparison between the log_2_ of gene expression ratios obtained from RNA-seq data and qRT-PCR

To validate the RNA-seq data, qPCR analysis of transcript abundance was performed of 17 randomly selected DEGs, including 12 that were up-regulated and 5 that were shown to be down-regulated in the unigene dataset (Additional file 2). As shown in Fig. 7, the fold change values obtained by qPCR were highly consistent with those based on the RNA-Seq data for all of the tested genes (Fig. 7), except for a difference in the expression patterns of two down-regulated genes at two time points. This result supports the reliability of the RNA-Seq analysis.

### Expression of genes involved in polyamine and ethylene metabolism

**Fig. 8:**
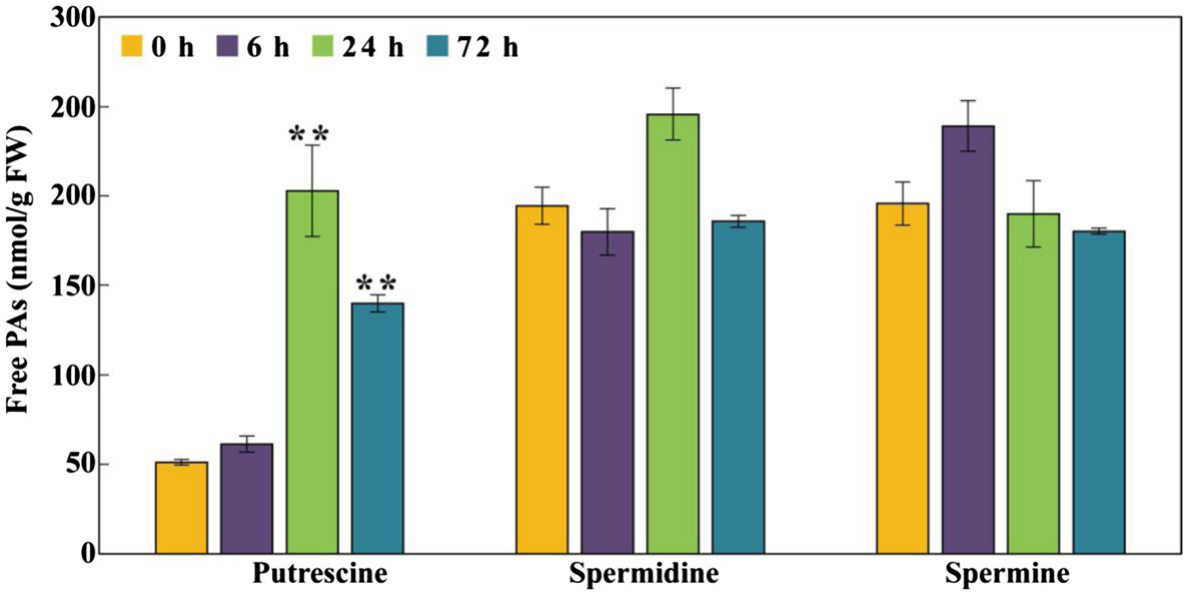
Quantification of free polyamines (PAs) of *Poncirus trifoliata* under cold treatment. Three types of free polyamines, including putrescine, spermine, and spermidine, are assessed by HPLC. The bars represent SE (n = 3). Asterisks show that the values are significantly when compared with the levels at the onset of cold treatment (**P < 0.01)

Polyamines (PAs), including diamine putrescine (Put), triamine spermidine (Spd) and tetramine spermine (Spm), are polycationic low molecular weight compounds that are ubiquitoius in most organisms. Several unigenes encoding polyamine biosynthetic enzymes, such as *ADC* (arginine decarboxylase), *ODC* (ornithine decarboxylase), *SAMDC* (S-adenosylmethionine decarboxylase), *SPDS* (spermidine synthase) and *SPMS* (spermine synthase) were identified, and of these *ADC* (Unigene25843_All), which encodes a key enzyme in Put biosynthesis, was highly expressed in the cold treated samples compared to the control samples. Meanwhile, the expression of *ODC* (Unigene17207_All, Unigene11015_All, CL6768.Contig1_All, CL9042.Contig1_All), an alternative enzyme involved in Put biosynthesis, showed little change in response to cold exposure. This finding indicates that the *ADC* pathway may be more important for the Put biosynthesis of *P. trifoliata* than the *ODC* one during cold stress. SAMDC is involved in the synthesis of spermidine and spermine by providing the aminopropyl moiety derived from a decarboxylation of *S*-adenosyl methionine (SAM). We observed that the *P. trifoliata SAMDC* unigene (Unigene20820_All) was expressed at higher levels after the low temperature treatment. Meanwhile, unigenes with homology to *SPDS*, *SPMS*, and *ACL5* showed no increase in expression levels during the cold treatment. Furthermore, the expression of SAM synthases (CL5314.Contig1_All, Unigene24811_All, and Unigene21190_All) was substantially induced. Furthermore, aminocyclopropane carboxylic acid (ACC) synthase (ACS) converts SAM to ACC, which is then oxidized into ethylene by ACC oxidase (ACO). Notably, an *ACS* gene (CL4785.Contig1_All) was down-regulated during cold stress, while expression of an *ACO* (CL6929.Contig1_All) gene was barely detectable over the entire time course. We also measured the content of free polyamines in the cold-treated samples. Put levels were progressively elevated under cold treatment, while levels of Spd and Spm underwent only minor changes with the exception of a slight increase of Spd at 24 h and Spm at 6 h (Fig. 8).

### Identification of transcription factors (TFs) in response to cold stress

**Fig. 9:**
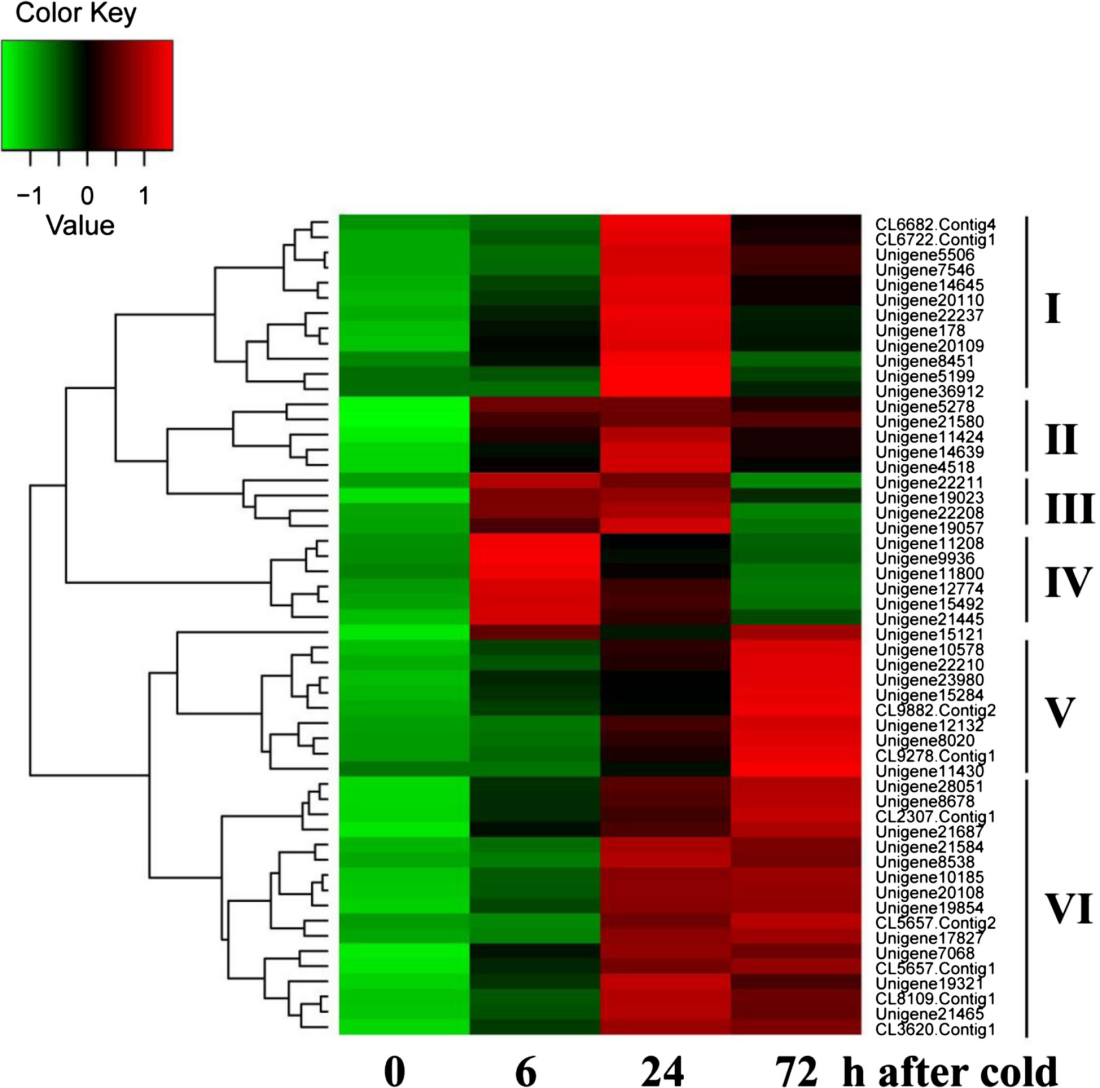
A heat map indicating expression patterns of transcription factors under cold stress. Columns and rows in the heat map represent samples collected at differenet time points of cold stress and common differenttially expressed TFs, respectively

We determined that the transcript abundance of 60 TFs exhibited highly dynamic changes in response to the cold treatment (Fig. 9), of which those encoding AP2/ERF domain-containing proteins constituted the largest group (43.3 %), followed by WRKY proteins (1.7 %), NACs (1.5 %), zinc finger proteins (ZFPs, 0.7 %), MYBs (0.7 %), and bHLHs (0.7 %). In addition, the expression of two heat shock transcription factors (Hsfs) (Unigene12132, CL9882.Contig2) was found to be higher following the cold treatments (Additional file 6). Transcript levels of Unigene12132_All were induced by 1.7, 3.8, and 4.5 folds at 6, 24 and 72 h in comparison with that of control, while CL9882.Contig2_All showed a progressive increase and was 2.8 fold higher than the control at 72 h.

The differentially expressed TFs were classified into six subgroups based on their expression patterns (Fig. 9). Subgroup I was characterized by genes showing low changes in transcript levels early during the cold treatment compared with the control plants, a peak of expression at 24 h and then a decrease at the 72 h time point. TFs in subgroup II showed a relatively high fold change at all three time points and five TFs belonged to this group, including three AP2/ERF domain-containing TFs, one NAC and one WRKY. The four TFs in subgroup III showed the highest expressional levels at 6 and 24 h, but lower levels at 72 h. The transcript abundance of TFs in subgroup IV, including four AP2/ERF domain-containing TFs, two zinc finger and a single WRKY, was greater at the early time point before declining, which suggests that they play roles in early transduction of the cold signal. TFs in subgroup V showed a peak in expression at 72 h, while TFs in subgroup VI, the largest subgroup, showed high levels of transcript abundance at 24 h and the levels remained high at 72 h.

Among the differentially expressed AP2/ERF TFs, four CBFs/DREBs-like unigenes (Unigene5199_All, Unigene36912_All, Unigene24431_All and Unigene14645_All) which were all clustered in subgroup I, and therefore had a similar expression pattern, were significantly induced by cold treatment. For example, Unigene24431_All, which was annotated as a *DREB2A*-like gene, had a RPKM value of 35.79, 139.60, 497.48 and 1085.57 in the 0, 6, 24 and 72 h samples. In addition, the expression of an ICE1-like TF (Unigene24033_All) was also found to be up-regulated in the cold-treated plants.

### Identification of hormone and signal transduction-related unigenes

The GO and KEGG enrichment analyses of the DEGs both highlighted signal transduction as being particularly affected by the cold treatment. More than 60 unigenes predicted to encode protein kinases were differentially expressed in trifoliate orange grown under the cold conditions (Additional file 7) and of these, genes encoding receptor-like protein kinases (RLKs) made up the largest group. More than 30 RLK genes were serine/threonine-protein kinase, three were wall-associated receptor kinases (Unigene20744_All, Unigene25402_All, Unigene22769_All), and one was a proline-rich receptor-like protein kinase (Unigene20499_All). Furthermore, the expression of *BRASSINOSTEROIDS INSENSITIVE* 1 (BRI1, CL3291.Contig2_All) was found to increase, while an annotated brassinosteroid LRR receptor kinase (Unigene19522_All) showed a decrease in transcript abundance under cold stress. Additionally, eight genes annotated as kinases were up-regulated. Mitogen-activated protein kinase (MAPK) cascades have been shown to play key roles in mediating stress-associated signaling pathway [33] and among the DEGs, a MAPK (CL8004.Contig1_All) and two MAPKKK (Unigene25596_All, CL3174.Contig_All) were up-regulated by the cold treatment. In addition, the expression of several kinases related to MAPK (mitogen-activated protein kinase) cascades was similarly induced.

We also observed that the expression of genes associated with biosynthesis or signal transduction of the phytohormones abscisic acid (ABA), gibberellin (GA) or ethylene showed substantial changes following cold temperatures. KEGG analysis of the DEGs suggested that carotenoid biosynthesis (ko00906), which is associated with ABA biosynthesis, was induced under cold condition. As an example, the expression of Unigene19385_All, which is predicted to encode a 9-*cis*-epoxycarotenoid dioxygenase (NCED), a key rate-limiting enzyme in ABA synthesis, was progressively up-regulated by ~3-4 folds at 24 h and 72 h, respectively, compared to the 0 h time point. Interestingly, the expression of a unigene (CL851.Contig21_All) encoding an ABA 8′-hydroxylase 1, a key enzyme in ABA catabolism, was rapidly up-regulated after 6 h of cold treatment, but its transcript abundance declined to the same expressional level as 0 h after 72 h. This apparent regulation of genes involved in ABA biosynthesis and catabolism suggests dynamic and multifaceted control of ABA content in response to stress. Also we also noted genes involved in ABA signaling pathway showed similar changes in expression. For example, three genes encoding protein phosphatase 2C (PP2C), CL6797.Contig2_All, CL6797.Contig1_All and Unigene24667_All, were up-regulated at 6 h, and four additional *PP2C* genes (CL3200.Contig4_All, CL9817.Contig1_All, Unigene24667_All, CL2118.Contig1_All and Unigene934_All) showed increase in transcript abundance after 24 h of cold stress. In contrast, three other *PP2C* genes (Unigene22810_All, Unigene21083_All, and Unigene23511_All) and a unigene (Unigene23445_All) encoding PYR/PYL (Pyrabatin Resistance/Pyrabatin Resistance 1-Like), ABA receptors, were down-regulated at 72 h.

Although expression of the genes associated with ethylene biosynthesis was generally down-regulated by the cold treatment, we identified unigenes related to ethylene meditated signaling that showed the opposite trend. This was exemplified by 11 ethylene signal transduction-related genes, three ethylene receptors (ETRs), a MAPK and seven ERFs, all of which were up-regulated by cold exposure. However, we also determined that a unigene (Unigene13110_All) encoding an Ein3-binding F-box protein (EBF1/2) (Unigene13110_All) was down-regulated.

**Fig. 10:**
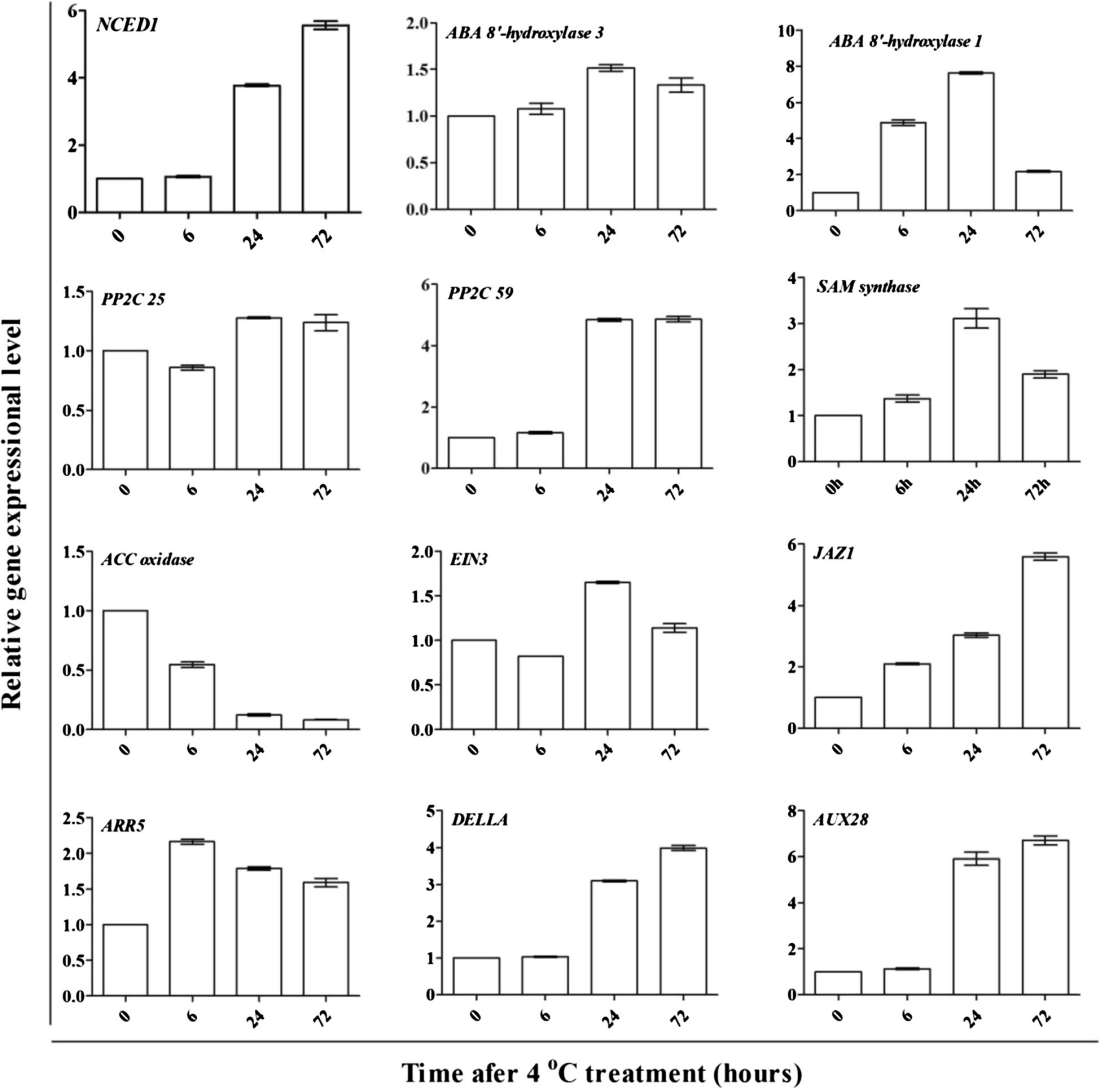
Expression patterns of genes involved in hormone metabolism and signal transduction. Expression of the selected genes was comfirmed by qPCR using samples treated at low temperature. The samples were different from those used for RNA-Seq. The bars represent SE (n = 3)

We also observed that the expression of six genes related to biosynthesis of the defense-related hormone jasmonic acid (JA) was upregulated: one encoding allene oxide cyclase (Unigene15722_All), two encoding 12-oxophytodienoate reductase 2 (CL5141.Contig4_All and CL5141.Contig2_All) and two encoding AMP dependent CoA ligase (CL2829.Contig1_All, Unigene24432_All). Furthermore, three genes encoding JAZ and five genes encoding MYC2, which have been shown to be involved in JA signaling pathway, were also significantly up-regulated by the cold conditions. The expression patterns of the genes involved in hormone metabolism and signal transduction were analyzed by qPCR (Fig. 10). Although the samples were different from those used for RNA-Seq, expression patterns were quite similar between the two methods, which further confirmed reliability of screening the DEGs.

Reactive oxygen species (ROS) are known to provide a signal that triggers cellular changes in response to altered environmental conditions [34]. The chloroplast is considered to be a major producer of ROS during abiotic stress and inhibited photosynthesis is a key factor that results in the accumulation of ROS [35]. In this study, transcript levels of unigenes associated with the ‘Photosynthesis-antenna proteins’ and ‘Photosynthesis’ GO categories, such as chlorophyll A/B binding protein (Unigene12186_All, Unigene4387_All, Unigene12405_All, Unigene18912_All, CL216.Contig1_All), chloroplast pigment-binding protein CP24 (CL9248.Contig2_All), cytochrome b6-f complex iron-sulfur subunit (Unigene1165_All), PSI reaction center subunit II (Unigene28504_All), and photosystem II oxygen-evolving enhancer protein (Unigene14933_All), decreased following the exposure treatment. Conversely, the abundance of transcripts corresponding to ROS receptor proteins or redox-sensitive transcription factors, such as Hsfs (Unigene12132_All, CL9882.Contig2_All) was up-regulated (Additional file 8). We noted that expression of the unigene CL8980.Contig1_All, which encodes MBF1c, a key regulator of thermotolerance in *A. thaliana* and a potential regulatory hub linking ROS signaling with pathogen and abiotic stress responses, increased in response to the cold treatment. It is known that ROS promotes cellular signaling thought a ROS-induced MAPK signaling pathway [33]. We identified several MAPKs in this study that were significantly up-regulated by the cold treatment, although their associations with ROS mediated-signaling have yet to be established.

Calcium serves as a second messenger and plays vital roles in a number of signaling pathways [36, 37]. In plants, oscillations in calcium levels are detected by calcium sensor proteins, such as calcium-related proteins, calmodulin and calmodulin-binding protein (CBP), calcium-dependent protein kinases (CDPKs) and calcium-regulated phosphatases, which relay signals and trigger downstream responses [38, 39]. In this study, we identified four genes (CL9078.Contig2_All, CL4696.Contig2_All, Unigene5810_All, CL653.Contig20_All) encoding CDPKs, one gene (Unigene22833_All) encoding a CBP and four genes (Unigene25450_All, Unigene26697_All, Unigene14626_All, and CL3238.Contig1_All) encoding CBL-interacting protein kinases (CIPKs), all of which were up-regulated by the cold treatment (Additional file 8).

## Discussion

### Illumina paired-end sequencing, assembly, and functional annotation

In this study, we performed an RNA-seq analysis of the *P. trifoliata* transcriptome using Illumina-based 2 × 90 bp paired-end reads sequencing. Illumina, a widely used NGS platform for transcriptome assembly, produces relatively shorter reads but generates higher transcriptome coverage at lower expenses compared with other platforms [40]. Approximately 6 Gb high-quality reads were obtained, which were *de novo* assembled into 77,292 unigenes. The average length of the assembled unigenes was 1112 bp and 55.5 % of the unigenes were longer than 1 kb, which indicates a high quality assembly that is likely to include many full-length cDNAs. In addition, 77.3 % of the assembled unigenes had homologs in at least one of the public databases that we searched, and 70.5 % of the unigenes had a homolog that was determined with a high probability score in three species: *V. vinifera* (31.8 %), *R. communis* (26.5 %) and *P. trichocarpa* (22.2 %). Significantly, only 274 unigenes aligned with previously reported *P. trifoliata* sequences, underlining the sparse gene coverage of this species in the Nr database. Taken together, these results suggested that our *P. trifoliata* EST dataset represents a valuable transcriptome resource for gene discovery and functional analysis.

### Analysis of DEGs that are responsive to cold temperatures

It has been well documented that plants can enhance their cold tolerance by modulating gene transcription, and examples of gene induction and repression in response to cold temperatures have been reported [41, 42]. In this study, 5549 *P. trifoliata* genes were differentially expressed as a consequence of the cold treatment. Of these, 462, 1631 and 2702 unigenes were up-regulated after 6, 24, and 72 h of cold treatment, respectively, while 138, 715 and 2475 unigenes were down-regulated, This predominance of gene induction during the cold stress has been found in other studies [11, 12]. It is notable that the number of DEGs progressively increased over the course of the cold treatment, indicating ongoing adaptations over an extended time. More than 28 % of the *P. trifoliata* DEGs had no annotated homologs in the Nr database and these may be specific to *P. trifoliata* or represent cold-responsive genes with homologs that have not been identified in previous studies with other plant species.

### Phytohormone signals under low temperature

The phytohormones ABA, GA and ethylene are known to play key roles in a wide range of adaptive responses to abiotic stresses [43]. In this study, the transcriptome data suggested that the expression of genes involved in signal transduction pathways related to several plant hormones were modified within 6 h of cold treatment. Moreover, increasing numbers of genes related to these pathways showed changes in expression over the 72 h of the cold treatment time course, suggesting a critical role of the hormone pathways in conferring trifoliate orange response to the cold stress. ABA is perhaps the most well characterized phytohormone in terms of integrating various stress signals and modulating downstream stress responses, although its involvement in cold responses is still debated [44]. It has been suggested that plant response to the cold stress may be ABA-independent, but there is increasing evidence to the contrary [45, 46]. NCED is a rate-limiting enzyme in ABA synthesis and earlier studies reported transcriptional regulation of *NCED* following abiotic stress [47]. Accordingly, in our analysis, we noticed that an *NCED* gene was up-regulated by approximately 3–4 folds after 24 and 72 h of cold treatment. Up-regulation of the *NCED* gene implies that synthesis of ABA might be elevated in trifoliate orange under cold. In addition, we detected the increased expression of *PP2C*, a key component in the ABA signaling pathway (Table 5). This result is consistent with studies of *P. euphratica* and *A. thaliana* [18, 48], but differs from an equivalent analysis of on *Lilium lancifolium* [13]*.* Thus, whether and how ABA mediated signaling is involved in the cold responses of trifoliate orange remains to be further established.

**Table 5:** Hormone-related genes that were differentially expressed during the cold treatment

Gene ID	Fold change			Annotation
	6 h vs.0 h	24 h vs. 0 h	72 h vs. 0 h	
ABA				
Unigene19385_All	0.82	3	3.8	9-cis-epoxycarotenoid dioxygenase (*NCED*) [*Citrus sinensis*]
CL927.Contig1_All	2.9	2.9	4	short chain alcohol dehydrogenase [*Ricinus communis*]
CL851.Contig21_All	5.7	4.1	0.51	ABA 8′-hydroxylase [*Citrus sinensis*]
Unigene33085_All	9.7	12.8	14.1	ABA 8′-hydroxylase [*Glycine max*]
Unigene15626_All	−1.1	2	4	ABA 8′-hydroxylase [*Vitis vinifera*]
CL6797.Contig1_All	11.2	11.6	9.9	protein phosphatase 2C 25 isoform 1 [*Vitis vinifera*]
CL3200.Contig4_All	7.9	12.2	10.5	protein phosphatase 2C 59 [*Vitis vinifera*]
CL9817.Contig1_All	3.4	4.1	2.7	protein phosphatase 2C (PP2C) [*Fagus sylvatica*]
Ethylene				
CL5314.Contig1_All	1.3	4.9	5	S-adenosylmethionine synthetase [*Litchi chinensis*]
Unigene20820_All	0.7	2.2	3	S-adenosylmethionine decarboxylase [x *Citrofortunella microcarpa*]
Unigene21338_All	−1	−1.7	−3.7	1-aminocyclopropane-1-carboxylate oxidase homolog 4-like [*Vitis vinifera*]
Unigene23036_All	0.4	1.5	2.6	probable 2-oxoglutarate/Fe(II)-dependent dioxygenase-like [*Vitis vinifera*]
Unigene28463_All	1.3	2.7	2.8	ethylene response 2 [*Citrus sinensis*]
CL9038.Contig1_All	11	10.4	9.6	ein3-binding f-box protein 4 [*Populus trichocarpa*]
Unigene5278_All	3.6	3.6	3.2	Ethylene-responsive transcription factor 1B, putative [*Ricinus communis*]
Unigene19854_All	2	3	3	Ethylene-responsive transcription factor, putative [*Ricinus communis*]
Unigene23980_All	1.1	1.3	2	AP2/ERF domain-containing transcription factor [*Populus trichocarpa*]
Unigene21687_All	2.8	3.2	3.6	ethylene responsive transcription factor 1a [*Prunus salicina*]
Unigene21465_All	1.1	2.1	1.9	AP2/ERF domain-containing transcription factor [*Populus trichocarpa*]
JA				
CL5141.Contig4_All	12.1	12.7	14.1	old-yellow-enzyme homolog [*Catharanthus roseus*]
Unigene15722_All	0.3	1.5	2	allene oxide cyclase 2 [*Glycine max*]
CL2829.Contig1_All	4	4.2	3.5	4-coumarate--CoA ligase-like 9-like [*Vitis vinifera*]
CL9506.Contig1_All	3.6	4.8	4.4	JAZ1 [*Vitis rupestris*]
Unigene17827_All	1.3	3.6	3.9	transcription factor bHLH041 [*Vitis vinifera*]
CL6722.Contig1_All	1.5	3.4	2.5	transcription factor bHLH30-like [*Glycine max*]
Unigene25537_All	0.9	1.8	1.9	transcription factor bHLH13 [*Vitis vinifera*]
Cytokinin				
Unigene25908_All	3.7	3.2	1.9	two-component response regulator ARR5-like [*Vitis vinifera*]
Unigene25909_All	1.5	2.3	2.2	two-component response regulator ARR5-like [*Vitis vinifera*]
Unigene20772_All	0.6	3.4	4.8	Glucan endo-1,3-beta-glucosidase precursor, putative [*Ricinus communis*]
CL8049.Contig1_All	0.6	1.5	0.8	glucan endo-1,3-beta-glucosidase-like protein 3-like isoform 2 [*Vitis vinifera*]
GA				
CL9460.Contig1_All	1.2	2.2	2.9	gibberellin 2-beta-dioxygenase 1-like [*Vitis vinifera*]
Unigene21338_All	−1	−1.7	−3.7	Gibberellin 20 oxidase [*Medicago truncatula*]
Unigene14999_All	3.3	4.7	5.7	GRAS family transcription factor [*Populus trichocarpa*]
Auxin				
CL7975.Contig1_All	4.2	6.1	6	cytochrome P450 83B1 [*Vitis vinifera*]
CL5737.Contig2_All	4.9	7	5.3	phenylalanine N-monooxygenase-like [*Vitis vinifera*]
Unigene22952_All	−0.4	−1	−1.9	protein AUXIN SIGNALING F-BOX 3 [*Vitis vinifera*]
CL9773.Contig2_All	0.9	4.9	6.2	IAA15 [*Solanum lycopersicum*]
Unigene20450_All	1.4	4.1	6	Auxin-responsive protein IAA1, putative [*Ricinus communis*]
Brassinosteroid				
Unigene19738_All	−0.1	4.6	4.6	cytochrome P450, putative [*Ricinus communis*]
Unigene33085_All	9.7	12.8	14.1	cytochrome P450, putative [*Ricinus communis*]
CL1502.Contig3_All	3.9	4.1	3.4	wall-associated receptor kinase-like 1-like [*Vitis vinifera*]
CL7113.Contig3_All	3.9	4.6	3.8	wall-associated receptor kinase-like 9-like [*Vitis vinifera*]
CL8664.Contig1_All	15.2	14.7	13.5	xyloglucan endotransglycosylase [*Cucumis sativus*]

Ethylene has been reported to participate in a wide range of cellular and developmental processes, and in both abiotic and biotic stress responses [49, 50]. The molecular mechanisms underlying perception and transduction of the ethylene signal and activation of hundreds of relevant genes have been extensively studied [51]. Ethylene signaling has been implicated in the cold stress response [52–55], but different studies have attributed it with either positive or negative effects. For example, the positive role of ethylene in cold stress was demonstrated in rice [52], tomato [53], winter rye [54] and *A. thaliana* [56], but Shi et al. [57] reported that ethylene signaling negatively influences freezing tolerance by repressing the expression of *CBF* and type-A *ARR* genes. The discrepancy may reflect differences in the species studied or to the growth conditions of the tissues used for the study [56]. In our study, we observed that the expression an ACC synthase gene (*ACS*, CL4785.Contig1_All) was repressed following cold stress. ACS is involved in ethylene synthesis and so its down-regulation may lead to reduced ethylene production (Fig. 10, Table 5). This result is consistent with an earlier study [52] and prompts us to propose that ethylene may be possibly a negative regulator of cold responses in trifoliate orange.

In addition to ABA and ethylene, other hormones may also play a role in cold stress response. The role of the growth-related hormone GA in the response to abiotic stress is becoming increasingly elucidated [58]. For example, DELLA proteins have been shown to suppress GA signaling and can promote GA degradation via the ubiquitin-proteasome [59]. Accordingly, exposure of *A. thaliana* seedlings to cold stress promoted DELLA accumulation and resulted in DELLA-mediated growth restriction [60]. We observed that the expression of a unigene (Unigene17855_All) encoding a DELLA protein was up-regulated, suggesting that degradation of GA may be expedited by cold stress. In this regard, the cold-exposed plants may slow their growth in order to conserve energy for adapting to the adverse environments. Taken together, these results suggest that diverse hormonal signals are involved in responses and the expression modulation of large numbers of downstream genes.

### ROS-mediated signals associated with cold response

ROS produced during abiotic stresses are known to be toxic to cellular functions when they accumulate to high levels; however, there is increasing evidence that at low levels ROS can mediate an array of signals [34]. Inhibition of photosynthesis is a significant cause of ROS production [35]. In this work, ‘photosynthesis-antenna proteins’ and ‘photosynthesis’ are two major GO terms that were associated with substantial change in transcript levels in response to cold stress. Interestingly, transcript levels of genes involved in these pathways were decreased after exposure to the cold treatment indicating the photosynthetic capacity of *P. trifoliata* was inhibited, in turn leading to ROS accumulation. The ROS signal is thought to be sensed by yet unidentified receptor proteins or redox-sensitive TFs, such as Hsfs [61]. In our study, two Hsf unigenes (Unigene12132_All, CL9882.Contig2_All) were differentially expressed in response to cold, but their potential roles in ROS signal sensing remain to be investigated. In addition, ROS signals are transduced to downstream targets by either a MAPK cascade or the action of TFs, such as zinc finger proteins (ZFPs), WRKYs and multiprotein bridging factor 1c (MBF1c) [62]. We observed in our study that the transcript levels of a MAPK (CL8004.Contig1_All) and two MAPKKKs (Unigene25596_All, CL3174.Contig_All) were up-regulated by the cold treatment, as were those corresponding to unigenes encoding several ZFP and WRKY proteins. We hypothesize that different components of the ROS signal transduction pathway are involved in mediating the cold stress response, and our dataset has revealed some potential candidates.

### Transcription factors involved in cold stress response

Numerous families of TFs are known to orchestrate the signals that are transduced when plants are challenged with various abiotic stresses, including the MYB, AP2/EREBP, WRKY, NAC, MYC and bHLH families, and individual members have been revealed to promote or suppress abiotic stress responses [63, 64]. In the current study, 54 putative TFs were found to be induced either at a given time point or during the whole course of the cold treatment. The largest group of the cold-inducible TFs belonged to the AP2/ERF family and was composed of 26 members. Of these, three unigenes (Unigene5199, Unigene14645 and Unigene36912) were annotated as *CBF/DREB* genes, which have been shown to play important roles in cold acclimation leading to freezing tolerance [5]. Our results therefore suggest that the CBF pathway is conserved in trifoliate orange responses to the cold stress. We also noticed that the cold stress induced the expression of TFs in the NAC (CL6682.Contig4, Unigene7546), bHLH (CL6722.Contig1, Unigene22237, Unigene8451), WRKY (Unigene5506, Unigene178), and ERF (Unigene20110) families. Earlier studies have shown that members of these families function in cold tolerance [65], but whether they work, independently or synergistically, to promote cold tolerance of trifoliate orange remains to be determined.

### Polyamine biosynthetic genes implicate in cold response

Upon exposure to various abiotic stresses, plants accumulate various metabolites that are thus considered as potential indices indicating the magnitude of stress tolerance [66]. As an example, polyamines (PAs) are viewed as important stress-related metabolites that can function to reduce damage associated with adverse environmental cues. The polyamines, being polycationic, stabilize cellular membranes through binding to anions or act as critical protective metabolites by mitigating osmotic stress [67]. A number of earlier studies reported that PA biosynthetic genes are up-regulated by cold conditions; and overexpression of these biosynthetic genes can confer cold tolerance [24]. In this study, we detected a change in the mRNA levels of annotated polyamine biosynthetic genes in response to cold stress, including *ADC* (Unigene25843_All) and *SAMDC* (Unigene20820_All), which encode two rate-limiting enzymes in the synthesis of putrescine, spermidine and spermine, respectively. The up-regulation of *ADC* gene coincides with the increase in the putrescine level in the cold-treated samples. However, *SAMDC* gene induction was not accompanied by a concurrent elevation of spermidine and spermine. These data suggest that synthesis of putrescine might be promoted, which may provide the plants to combat the cold stress.

## Conclusions

In this study, a *P. trifoliata* transcriptome dataset comprising 77,292 predicted transcripts was generated by high-throughput sequencing and dynamic changes in gene expression under cold treatments were observed. A large number of cold-responsive genes were revealed, including those encoding TFs, ROS and hormone signaling elements, and enzymes associated with the synthesis of protective metabolites. The transcriptome and digital expression profiling of *P. trifoliata* provide a valuable resource for functional evaluation of the cold-responsive genes, which are assumed to hold great potential for genetic engineering of cold tolerance.

## Methods

### Plant materials and stress treatments

Three-month-old trifoliate orange (*Poncirus trifoliata* (L.) Raf.) seedlings were collected from an experimental nursery at Huazhong Agricultural University. The plants were carefully and extensively washed and placed in glass beakers of tap water and kept for 5 d at 25 °C in a growth chamber. In order to promote the expression of stress-responsive genes, the seedlings were treated with different stresses, including cold, high salinity, and drought. For the cold treatment, the plants were placed at 4 °C and the leaves were harvested immediately (0 h time point) or after 6, 24 or 72 h. The salinity treatment was applied by placing the seedlings in 200 mM NaCl solution for same time periods as the cold treatment. For the drought treatment, the seedlings were placed on filter paper and dehydrated at ambient room temperature and the whole plants were collected at 0, 0.5, 3, and 6 h. For each time point, at least 20 seedlings were used, and the collected samples were immediately frozen in liquid nitrogen after sampling and stored at −80 °C until use for further use. In addition, a new set of seedlings were treated at 4 °C for 0, 6, 24 or 72 h; the leaves were sampled for analysis of expression patterns of several genes involved in hormone metabolism and signal transduction.

### RNA extraction

Total RNA was extracted using an RNAiso Plus kit (TaKaRa, Dalian, China) according to the manufacturer's instructions. The concentration and quality of total RNA was evaluated with a NanoDrop^TM^ 2000 UV–vis Spectrophotometer (Thermo Scientific, Waltham, MA, USA). For transcriptome sequencing, equal amount of total RNA from the stress-treated samples were pooled.

### cDNA library construction and RNA-sequencing

The pooled cDNA library was constructed using an mRNA-Seq assay for paired-end transcriptome sequencing. In brief, poly(A) mRNA was enriched from 20 μg of total RNA using oligo(dT) magnetic beads, and sheared into short fragments (200–700 bp) with fragmentation buffer. The short mRNA fragments were synthesized into double-strand cDNAs using random hexamer primers, and purified with a QiaQuick PCR Purification Kit (Qiagen, CA, USA). Then cDNAs were washed with EB buffer (10 mM Tris–HCl, pH 8.5) for end-repairing and poly(A) addition. Illumina paired-end sequencing adapters were then ligated to the ends of the 3′-adenylated cDNA fragments. Agarose gel electrophoresis was carried out to select fragments of a suitable size that was enriched by PCR amplification to construct the cDNA library. The library was sequenced by BGI (Shenzhen, China) on an Illumina HiSeq^™^ 2000 platform.

### Bioinformatics analysis of the transcriptome

Raw reads generated by the Hiseq^TM^ 2000 were filtered to remove low quality reads (reads containing more than 50 % bases with Q-value ≤20), adaptor-containing reads, and reads containing more than 5 % ambiguous nucleotides. After the preprocessing, >5 Gb filtered short reads, termed ‘clean reads’, were obtained. These were then *de novo* assembled using Trinity to generate a collection of non-redundant unigenes. To this end, the reads with overlapping sequences were merged to generate longer contiguous sequences (contigs), and the reads were then mapped back to the contigs. The relation and distance among these contigs were determined based on paired-end reads, which enabled the detection of contigs from the same transcripts as well as the distances between these contigs. The contigs were then assembled using Trinity to identify the sequences without end extension, which were defined as unigenes. TIGR Gene Indices clustering tools (TGICL) [68] was used to eliminate redundancy and further assemble the unigenes. The unigenes displaying >70 % sequence similarity were grouped into a cluster and the prefix CL was assigned to each cluster, while those from the other group was considered as singletons with the prefix unigene.

All non-redundant transcripts were subjected to BLASTx alignment (*E*-value < 10^−5^) against public protein databases, including Nr, Swiss-Prot, KEGG and COG. The assembled unigenes were annotated using Blast2GO to generate GO terms according to molecular function, biological process and cellular component, based on the best BLASTx hit from the NR database. Unigenes without an annotation were analyzed by ESTScan to predict coding regions and sequence direction. Metabolic pathways of the unigenes were determined according to Kyoto Encyclopedia of Genes and Genomes (KEGG) database [69].

### Analysis and mapping of DEG reads

Four cDNA libraries from trifoliate orange treated with cold for 0, 6, 24, and 72 h were sequenced and the raw data were processed as described above. The short cleaned reads were mapped to the assembled unigenes using SOAPaligner/soap2 software. The read counts, indicating unigene expression, in different libraries were normalized as RPKM (reads per kilobase per million mapped reads) as described by Mortazavi et al. [70]. Differentially expressed genes (DEGs) were identified using the R package. The DEGs were also annotated using the GO database, and the numbers of DEGs in each GO term were calculated. KEGG pathway analysis of the DEGs was also performed to identify the associated biochemical and signal transduction pathways. The data of this study have been deposited in NCBI’s Gene Expression Omnibus (GEO) under an accession number GSE67439 and are accessible through GEO Series (http://www.ncbi.nlm.nih.gov/geo/query/acc.cgi?acc=GSE67439).

### Quantitative real-time RT-PCR analysis

Quantitative real-time RT-PCR (qPCR) was performed with a Roche LightCycler 480 Real-Time System (Roche, Switzerland) to examine expression patterns of the selected unigenes. Each reaction contained 5 μl of 2x QuantiFast SYBR Green PCR Master Mix (Qiagen, Hilden, Germany), 1 μl of cDNA, and 1 μM of gene-specific primers in a final volume of 10 μl. The PCR reactions were performed under the following conditions: 95 °C for 5 min, followed by 45 cycles of 95 °C for 15 s, 58 °C for 20 s and 72 °C for 20 s. Relative expression levels of each gene were calculated using the 2^-ΔΔCt^ algorithm by normalizing to expression of expression of the *P. trifoliata Actin* gene, which was used as an internal control. Four technical replicates were used for each sample and the data are shown as means ± standard errors (SE) (n = 3). The source of variation resulted from the technical errors, such as operational approach, equipment and reagent. The primer sequences used for qPCR are listed in Additional file 9.

### Quantification of free PAs by high-performance liquid chromatography (HPLC)

Free polyamines were measured as described by Fu et al. [71] with some modifications. Briefly, leaf samples were ground into powder in liquid nitrogen with a pestle and mortar and ~0.1 g of powder was homogenized in 1 ml 5 % pre-cooled perchloric acid (PCA) containing 0.5 g/L dithiothreitol (DTT), and kept on ice for 30 min, followed by centrifugation at 13,400 × g for 15 min at 4 °C. This extraction was then repeated, and the supernatants were combined, and 1 ml was mixed with 50 μl of 1 M hexamethylene diamine, 1 ml of 2 M NaOH and 10 μl of benzoyl chloride. The mixture was vortexed for 30 s and placed in a water bath for 30 min. Subsequently, 2 ml of saturated NaCl and 2 ml of diethyl ether were added to the mixture and mixed well by vortexing before centrifugation at 6900 × g for 5 min. One ml of the supernatant was dried in a concentrator (Labconco, USA) and dissolved in 500 μl of methanol (Fisher, USA). The solution was filtered through 0.22 μm filter membrane and 20 μl was analyzed using an Agilent 1200 HPLC system (USA). The polyamines were determined in triplicate.

## Additional files

Additional file 1:
**KEGG mapping of the** ***P. trifoliata*** **transcriptome (XLSX).**
Additional file 2:
**Differentially expressed genes (DEGs) between the cold treatment (6 h, 24 h and 72 h) and control (0 h, untreated plants).** RPKM: reads per kilobase per million mapped reads. FDR: false discovery rate (XLSX). Additional file 3:
**Saturation evaluation of different gene expressions (A) and output of cleaned reads (B) (PDF).**
Additional file 4:
**Scatter diagram showing the distribution of up-regulated differentially expressed genes (DEGs) in red and down-regulated DEGs in green (PDF).**
Additional file 5:
**Significantly enriched GO terms amongst the differentially expressed genes (XLSX).**
Additional file 6:
**List of 60 differentially expressed genes (DEGs) encoding transcription factors (XLSX).**
Additional file 7:
**Differentially expressed genes (DEGs) encoding protein kinases and DEGs involved in phytohormone metabolism and signaling transduction pathway (XLSX).**
Additional file 8:
**A list of some of the important differentially expressed genes (DEGs) involved in photosynthesis, ROS and Ca** ^**2+**^ **-mediated signal transduction (PDF).**
Additional file 9:
**Primers used for qPCR analysis (PDF).**

## Abbreviations

ACS: Aminocyclopropane carboxylic acid (ACC) synthase
ADC: Arginine decarboxylase
CA: Cold acclimation
CBF: C-repeat (CRT)-binding factor
CDSs: Coding sequences
COG: Clusters of orthologous groups
COR: Cold-regulated
DEGs: Differentially expressed genes
DREB1: Dehydration-responsive element-binding factors 1
FPKM: Fragments per kb per million fragments
GO: Gene ontology
HPLC: High-performance liquid chromatography
KEGG: Kyoto encyclopedia of genes and genomes
MAPK: Mitogen-activated protein kinase
qPCR: Quantitative real-time RT-PCR
ODC: Ornithine decarboxylase
ROS: Reactive oxygen species
SAMDC: *S*-adenosylmethionine decarboxylase
SPDS: Spermidine synthase
SPMS: Spermine synthase
TF: Transcription factor
