# Supplementary material for: Deep sequencing-based characterization of transcriptome of trifoliate orange (Poncirus trifoliata (L.) Raf.) in response to cold stress
Source: BMC Genomics. 2015 Jul 29;16(1):555. doi: 10.1186/s12864-015-1629-7 (PMC4518522; doi:10.1186/s12864-015-1629-7)
Supplement: Additional file 3: — Saturation evaluation of different gene expressions (A) and output of cleaned reads (B) (PDF). [file 12864_2015_1629_MOESM3_ESM.pdf]

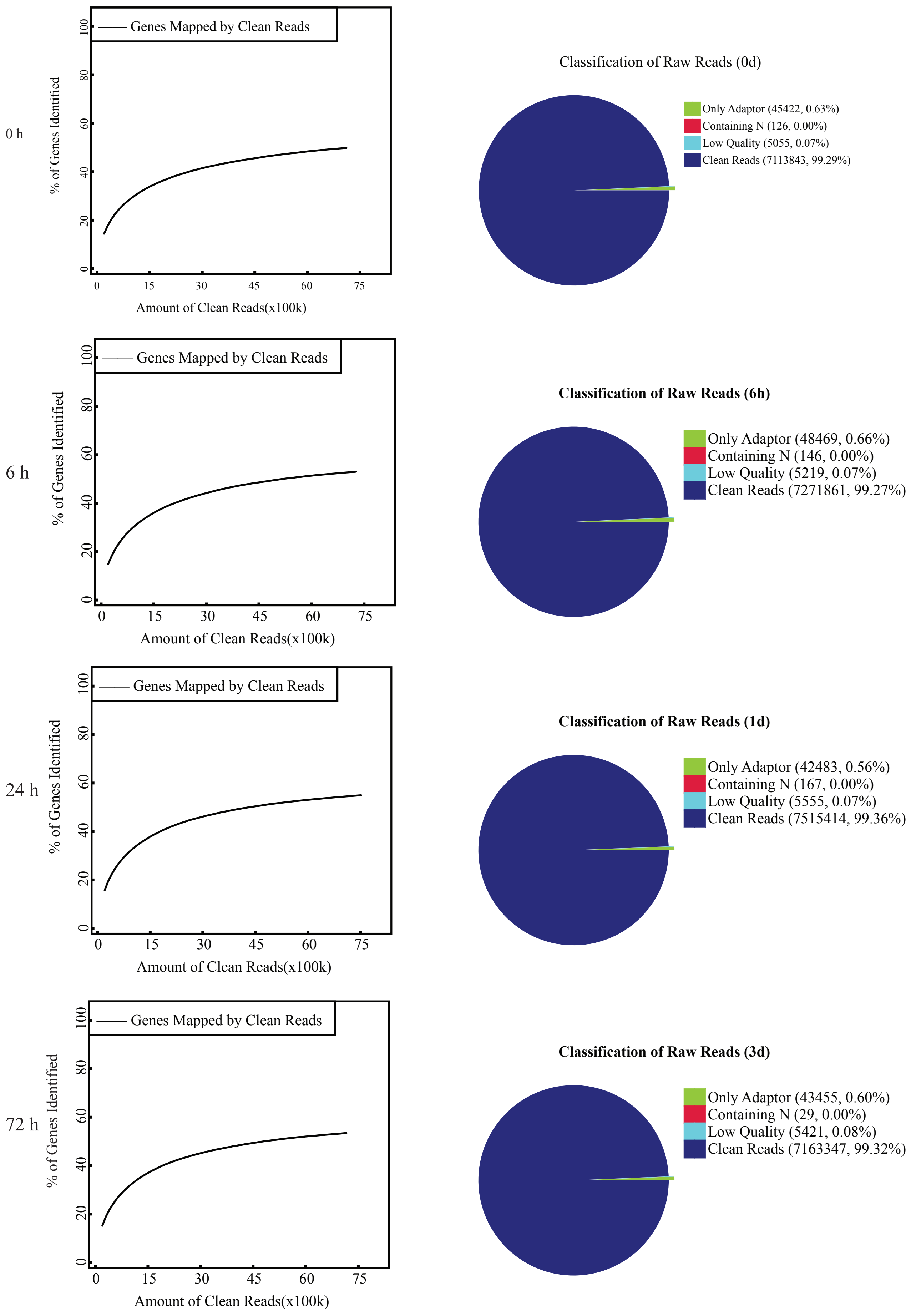

Additional file 2. Saturation evaluation of different gene expression (A) and output of clean reads (B).
