# Supplementary material for: Deep sequencing-based characterization of transcriptome of trifoliate orange (Poncirus trifoliata (L.) Raf.) in response to cold stress
Source: BMC Genomics. 2015 Jul 29;16(1):555. doi: 10.1186/s12864-015-1629-7 (PMC4518522; doi:10.1186/s12864-015-1629-7)
Supplement: Additional file 8: — A list of some of the important differentially expressed genes (DEGs) involved in photosynthesis, ROS and Ca 2+ -mediated signal transduction (PDF). [file 12864_2015_1629_MOESM8_ESM.pdf]

Additional file 9. A list of some of the important differentially expressed genes.

Gene ID	Gene description	Fold change		
		6h vs. 0h	24h vs. 0h	72h vs. 0h
**Photosynthesis-related genes**				
Unigene12186_All	Chlorophyll A/B binding protein	0.28	-0.35	-1.12
Unigene4387_All	Chlorophyll A/B binding protein	-0.08	-0.50	-1.20
Unigene12405_All	Chlorophyll a-b binding protein	-0.19	-0.65	-1.02
Unigene18912_All	Chlorophyll A/B binding protein	-0.14	-0.60	-1.22
CL216.Contig1_All	Chlorophyll A/B binding protein	-0.19	-1.06	-1.15
CL9248.Contig2_All	Chloroplast pigment-binding protein CP24	-1.66	-2.97	-6.60
Unigene1165_All	Cytochrome b6-f complex iron-sulfur subunit	-1.74	-3.61	-4.54
Unigene28504_All	PSI reaction center subunit II	-0.28	-1.08	-1.81
Unigene14933_All	Photosystem II oxygen-evolving enhancer protein	0.10	-0.71	-2.07
**ROS signal transduction**				
Unigene12132_All	Heat shock DNA binding protein	1.71	3.78	4.45
CL9882.Contig2_All	HSF domain class transcription factor	1.34	1.74	2.75
CL8980.Contig1_All	Multiprotein-bridging factor 1c	-0.37	0.78	1.54
**Ca^{2+} signal transduction**				
CL9078.Contig2_All	Calcium-dependent protein kinase	3.08	4.37	4.31
CL4696.Contig2_All	Calcium-dependent protein kinase	3.70	3.60	3.21
Unigene5810_All	Calcium-dependent protein kinase CDPK1	0.76	2.39	2.36
CL653.Contig20_All	Calcium-dependent protein kinase	0.72	2.22	2.40
Unigene22833_All	Calmodulin-binding protein	3.03	2.97	1.14
Unigene25450_All	CBL-interacting serine/threonine-protein kinase	0.72	2.58	2.27
Unigene26697_All	CBL-interacting protein kinase 9	0.42	1.36	1.01
Unigene14626_All	CBL-interacting serine/threonine-protein kinase	0.03	1.36	2.64
CL3238.Contig1_All	CBL-interacting protein kinase 4	1.80	2.77	3.25
