# Supplementary material for: Deep sequencing-based characterization of transcriptome of trifoliate orange (Poncirus trifoliata (L.) Raf.) in response to cold stress
Source: BMC Genomics. 2015 Jul 29;16(1):555. doi: 10.1186/s12864-015-1629-7 (PMC4518522; doi:10.1186/s12864-015-1629-7)
Supplement: Additional file 4: — Scatter diagram showing the distribution of up-regulated differentially expressed genes (DEGs) in red and down-regulated DEGs in green (PDF). [file 12864_2015_1629_MOESM4_ESM.pdf]

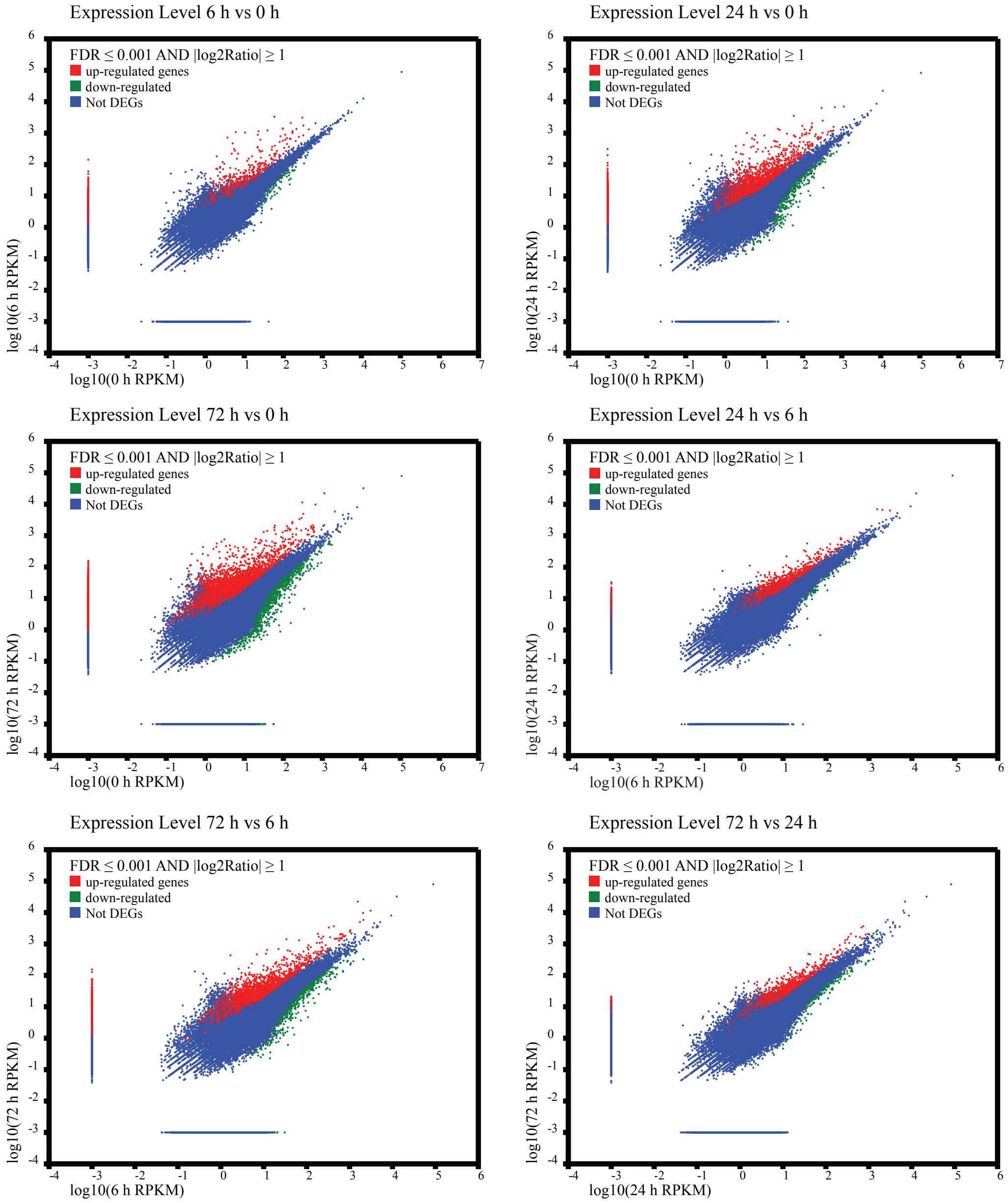

Additional file 3. Scatter diagram of the up-regulated DEGs (shown in red), down-regulated genes (shown in green), and unaltered genes(shown in blue).
