# Supplementary material for: Deep sequencing-based characterization of transcriptome of trifoliate orange (Poncirus trifoliata (L.) Raf.) in response to cold stress
Source: BMC Genomics. 2015 Jul 29;16(1):555. doi: 10.1186/s12864-015-1629-7 (PMC4518522; doi:10.1186/s12864-015-1629-7)
Supplement: Additional file 9: — Primers used for qPCR analysis (PDF). [file 12864_2015_1629_MOESM9_ESM.pdf]

Additional file 8. List of primers used in qRT-PCR

1. Primers were used in Validation of DEG results.

Unigene ID	Annotation	Primer name	Sequence
ACTIN	ACTIN	ACTIN-F	CCAAGCAGCATGAAGATCAA
		ACTIN-R	ATCTGCTGGAAGGTGCTGAG
Unigene20109	AP2/ERF	Unigene20109-F	TCCTCAAAGCGACCGAGATTC
		Unigene20109-R	CGCCGTCCAATCTTGAAAT
Unigene8451	Transcription factor bHLH113	Unigene8451-F	TTGAATGTTGAAGTGGCGTGT
		Unigene8451-R	GTCCATTTTCTCTCTTCTTTCCAG
Unigene11424	WRKY transcription factor 6	Unigene11424-F	CGTTCTTTTCCCAACACTGCTA
		Unigene11424-R	CAAGAGTCCCTTCCAATGCTGTC
Unigene21580	NAC domain protein	Unigene21580-F	CGATAAGGAGGTCCAAAGTGTG
		Unigene21580-R	ATCCATCTGATACTGAACTTGCG
Unigene19023	CHHH-type zinc finger protein 29-like	Unigene19023-F	TCTTTGCTCACAAGCCCGA
		Unigene19023-R	TTCTGCCACAAGTTTCCGC
Unigene8228	Wall-associated receptor kinase-like 4	Unigene8228-F	AGGGTGTGCATCAATAGAAAAAGC
		Unigene8228-R	AAGTGGCATTTGAATTAGGGGG
Unigene23840	Glucose-6-phosphate	Unigene23840-F	ACTTCGAACCATCTCGGCTG
		Unigene23840-R	TTGGCCTTCCGTCGTCATAG
Unigene31782	Ubiquitin-conjugating enzyme protein	Unigene31782-F	TGCCCATATGAGATTGGTCC
		Unigene31782-R	GCATGTCTAGGTATGCCACC
Unigene19064	Serine/threonine-protein kinase	Unigene19064-F	GACTTGAATGTGGCGACGTG
		Unigene19064-R	AGAGAAGCGTCCAACTGCTC
CL5510.Contig1	low-temperature-induced protein	CL5510.Contig1-F	AAGGTGCTGTAGGTTCGTGG
		CL5510.Contig1-R	CGCTCCCCAGTAGTGTTGTT
Unigene25843	Arginine decarboxylase	Unigene25843-F	AGTCATTGACATCGGGGGCG
		Unigene25843-R	GCTTGAACAACGGCAGAGGC
Unigene178	WRKY transcription factor 46	Unigene178-F	GCGCACTTGCACAAATTTCC
		Unigene178-R	GGGTGCACCTGTAATACCCT
CL8401.Contig1	Serine/threonine-protein kinase	CL8401.Contig1-F	CTGATCTCAGCTGTGCTGTTT
		CL8401.Contig1-R	CACTAATTCGACTGCATTATTTGGA
Unigene25373	CCCH-type zinc finger protein 18	Unigene25373-F	TAACCTTCCTGAGAGCCCGT
		Unigene25373-R	TCCTTCATTCGGGATGCACT
CL3540.Contig1	Alcohol dehydrogenase-like 2	CL3540.Contig1-F	TGTGGAGAGTGTAGGAGGAGG
		CL3540.Contig1-R	CCCTGAACCTGTTCGTTCCA
CL4044.Contig2	Predicted protein	CL4044.Contig2-F	GATCTAAGCCCTCACTGCCG
		CL4044.Contig2-R	ATGATGCAAAATCGGTGGCG
Unigene6992	ERF034-like	Unigene6992-F	CAAGCATCGGACCTGCAGTA
		Unigene6992-R	CCTTTGATGGCTAAAGCGGC

*F represent forward primer sequence (5′-3′), R represent Reverse primer sequence (5′-3′).

2. Primers were used in qRT-PCR of hormone-related genes.

Gene ID	Annotation	Primer name	Sequence
CL3200.Contig4	*PP2C 59*	CL3200-F	CAAAAAGCTTACCCCAGCCG
		CL3200-R	CCACATTCCAAACAGAGGGTG
CL5314.Contig1	*SAM synthase*	CL5314-F	CTTACGCTATTGGTGTGCC
		CL5314-R	GAAGTTTCTCCCACTTGAG
CL6797.Contig1	*PP2C 25*	CL6797-F	AGCCGGAGACGGAGATGAA
		CL6797-R	CTGGTCAAAGACAGCTACAG
CL851.Contig21	*ABA 8'-hydroxylase 1*	CL851-F	GTTTCTTTGCCAAAAGGTGAAG
		CL851-R	TTTTCGCCTTACGGCACCTC
Unigene33085	*ABA 8'-hydroxylase 3*	33085-F	ACATTGGGAAGATTCTCGGTGA
		33085-R	CATCCACTGAGAGGGGCTCA
CL9038.Contig1	*EIN3*	CL9038-F	TGCAAAACCCATCAAACAAGGC
		CL9038-R	CTACCGTGGTACGGTCGTTT
CL9506.Contig1	*JZA1*	CL9506-F	ACTACCTTCGCAAGTAGCCG
		CL9506-R	GTCCTGAATGATCCACACATT
Unigene14999	*DELLA*	14999-F	ATGCGTGCATGTTCAGCTCC
		14999-R	TCAAGTCCAGGATGAAATTGC
Unigene19385	*NCED1*	19385-F	ACAGAGGAGCAGATTCCGGG
		19385-R	CCTTATGGCTTCCACGGGAC
Unigene20450	*AUX28*	20450-F	CGATGCAATGGCCTCGACT
		20450-R	AGCTCCATCAACTGCCACC
Unigene20820	*SAMDC*	20820-F	CTCAGGATGGTTTGCGGACG
		20820-R	TCTTGGTCTCTGAATCGAGG
Unigene21338	*ACC oxidase*	21338-F	TCTGCCGATACTGAATGGC
		21338-R	AACACTCGATGCTCCACGCT
Unigene25908	*ARR5*	25908-F	TTTTGTGCAGGGAAGAGGCAAT
		25908-R	TGAGTGGTGGGGTTTGACTGT

*F represent forward primer sequence (5′-3′), R represent Reverse primer sequence (5′-3′).

3. Sequence of *ACTIN* gene

>ACTIN

TATTCCTTGGCTCTTCCGCTTCTCTCACTACTACATTTTTAATCTCTCGGCAG
TACCAAATACAGAGCGATAGAGAGAATCGACACTCTCAGCTAGACTCCATC
TTCGAATTTTTCTCTCGTCGTCTCTTCAATCTAACTTTTATAAAAAATGGCCG
ATGCTGAGGATATTCAACCCCTTGTTTGTGACAATGGAACTGGAATGGTGA
AGGCTGGATTTGCTGGAGATGATGCTCCAAGGGCAGTATTCCCTAGTATTGT
TGGTAGGCCTCGACACACTGGAGTCATGGTTGGAATGGGGCAGAAGGATG
CCTATGTTGGTGATGAGGCCCAATCGAAAAGAGGTATTCTTACTTTGAAATA
CCCAATTGAGCACGGTATTGTAAGCAACTGGGATGATATGGAAAAGATCTG
GCATCATACCTTCTACAACGAGCTTCGTGTTGCTCCTGAAGAGCACCCAGT
GCTTCTCACTGAGGCACCTCTTAACCCCAAGGCCAACAGAGAGAAGATGA
CCCAAATTATGTTTGAAACTTTCAATGTGCCAGCCATGTACGTTGCCATCCA
GGCTGTTCTATCTCTTTATGCCAGTGGTCGTACAACTGGTATTGTGCTGGAT
TCTGGTGATGGTGTGTCTCATACTGTACCAATTTATGAGGGATATGCCCTTCC
TCATGCCATTCTTCGTTTGGACTTGGCTGGTCGTGATCTCACTGATGCATTG
ATGAAGATTCTTACTGAGAGAGGTTACATGTTTACCACCACAGCCGAACGG
GAAATTGTCCGTGACATGAAGGAGAAGCTTGCTTATGTTGCCCTAGACTAT
GAGCAAGAACTTGAGACTGCCAAGAGCAGCTCATCTGTTGAGAAGAACTA
TGAGCTGCCTGATGGCCAGATCATCACCATTGGAGCTGAAAGATTCCGTTG
CCCAGAAGTTCTGTTCCAGCCATCTCTCATCGGAATGGAAGCTGCTGGTATT
CATGAGACTACCTACAACTCCATCATGAAGTGTGATGTGGATATTAGAAAG
GATCTGTATGGTAACATTGTTCTCAGTGGGGGTTCAACTATGTTCCCCGGTA
TTGCCGACCGTATGAGCAAGGAAATCACAGCACTTGCTCCAAGCAGCATG
AAGATCAAGGTCGTGGCTCCACCAGAGAGAAAATACAGTGTTTGGATTGG
AGGATCAATCCTTGCATCCCTCAGCACCTTCCAGCAGATGTGGATCTCAAA
GGGCGAGTATGATGAGTCTGGTCCATCCATTGTCCACAGGAAATGCTTCTA
AGTTCTTTAAGTGCTTGATGGGTGAGTTCTTTTTTTCCATTTAGTTGGTTTTT
TGTGTCTCGTTTCGCGAACTCAAGTTGGTTGACATGGAGAAGTGCTAAGGT
TGGGGCCATTGAAGGAGGCATATCTCGATATTGATGTTATTATACAAGCTTCT
TACAGCGTTTGAGGGGGGCTTATTTCTGTACTCAACCGTCTTCCTTGCCTTG
CATTCATCTGGTGTTCAACCAAGTCTTTTTTCTTTTTTTTCCTTTTTTTTTTTT
AAAGTAGGATGTTTGTAGCTGGAGATGAAGAGTGGTTGTGATGCTTTTCTAT
TTCATTTTCTAAATTTTCATATTTGAAGGTTTTTTTTCTCCCCCTCTCAAAAG
GAACATTAATGTTAATAGCTATTGTATGAGAAAACTTTAATTAGTGTCAGCTT
GCAGTCATAAATTTGGTTATTAAAATATATTTAATACTTGTTTGAGAGCTTTC
AA
